# Glia-Driven Brain Circuit Refinement Is Altered by Early-Life Adversity: Behavioral Outcomes

**DOI:** 10.3389/fnbeh.2021.786234

**Published:** 2021-12-02

**Authors:** Katrina A. Milbocker, Taylor S. Campbell, Nicholas Collins, SuHyeong Kim, Eva A. Smith, Tania L. Roth, Anna Y. Klintsova

**Affiliations:** Department of Psychological and Brain Sciences, University of Delaware, Newark, DE, United States

**Keywords:** early-life adversity, synaptic pruning, myelination, hippocampus, prefrontal cortex, behavioral impairment

## Abstract

Early-life adversity (ELA), often clinically referred to as “adverse childhood experiences (ACE),” is the exposure to stress-inducing events in childhood that can result in poor health outcomes. ELA negatively affects neurodevelopment in children and adolescents resulting in several behavioral deficits and increasing the risk of developing a myriad of neuropsychiatric disorders later in life. The neurobiological mechanisms by which ELA alters neurodevelopment in childhood have been the focus of numerous reviews. However, a comprehensive review of the mechanisms affecting adolescent neurodevelopment (i.e., synaptic pruning and myelination) is lacking. Synaptic pruning and myelination are glia-driven processes that are imperative for brain circuit refinement during the transition from adolescence to adulthood. Failure to optimize brain circuitry between key brain structures involved in learning and memory, such as the hippocampus and prefrontal cortex, leads to the emergence of maladaptive behaviors including increased anxiety or reduced executive function. As such, we review preclinical and clinical literature to explore the immediate and lasting effects of ELA on brain circuit development and refinement. Finally, we describe a number of therapeutic interventions best-suited to support adolescent neurodevelopment in children with a history of ELA.

## Introduction

Perinatal neurodevelopment represents the period with the greatest neuroplastic potential. Orchestration of several programmed cellular processes during early critical periods is necessary for homeostatic brain development as it leads to the emergence of adaptive behaviors that allow an organism to interact harmoniously with its environment. Brain tissue growth is marked by massive neuro- and gliogenesis which peaks during late embryogenesis. Following cellular proliferation and neuronal differentiation, axonal connections are established through synaptogenesis to facilitate neurotransmission. Neurotransmission during the brain growth spurt is primarily excitatory in nature and results in an over-abundance of working synapses which are supported by astrocytic processes at the tripartite synapse. Neural circuits imperative for survival and early-life functions such as suckling, vision, and audition are almost immediately myelinated by oligodendrocytes. However, redundant connections are phagocytosed by microglia during the phase of synaptic pruning which occurs during a sensitive period in late childhood and early adolescence, closing the perinatal period of elevated neuroplastic potential. The refined neural circuitry is then further myelinated to strengthen and optimize salient brain connections which are required for efficient adult cognitive processing. As such, glial proliferation and function are major constituents of proper neurodevelopment and are imperative for sustaining extraordinary neuroplastic potential during the perinatal and adolescent periods.

Mammalian neurodevelopment is a gradual process that continues well into the third decade of life in humans and, consequently, is susceptible to experience-driven alteration (Pujol et al., 1993; Lebel and Beaulieu, 2011; Miller et al., 2012). In this review, we discuss how several forms of early-life adversity (ELA), experiences that deviate from the expected and cause stress, lead to immediate changes in critical period onset and progression and cause downstream deficits in sensitive period ontogeny. ELA exposure, also referred to as exposure to “adverse childhood experiences (ACE),” has a high prevalence in the US and is comorbid with other environmental effectors including socioeconomic status, nutrition, and prenatal drug exposure which lead to several negative behavioral outcomes and the emergence of neuropsychiatric disorders later in life (Hambrick et al., 2019).

The amount and timing of ELA exposure are key factors which determine the effect of a stressful event on behavior and the potential emergence of a neuropsychiatric disorder in adolescence or young adulthood. The effect of ELA on brain development is modulated, in part, by the Stress Hyporesponsive Period (SHRP) which prevents an infant’s hypothalamic-pituitary-adrenal (HPA) axis from becoming overstimulated and producing an abundance of glucocorticoids during critical periods in neurodevelopment which may be damaging. The SHRP begins about 36 h after parturition and reduces basal corticosterone levels from between 15 and 25 μg/100 ml during late gestation to 1–3 μg/100 ml which is sustained until the end of the brain growth spurt around postnatal day 15 in the rat (Sapolsky and Meaney, 1986). A similar period is observed during the human brain growth spurt (Loman and Gunnar, 2010). Short and predictable periods of early-life stress do not lead to HPA axis over-activation, and instead result in the development of adaptive behaviors as early exposure to low levels of glucocorticoids induces HPA axis maturation (Popoli et al., 2011; Guan et al., 2020). However, ELA often presents as a prolonged or unpredictable stressor that has the potential to lower the SHRP threshold and elevate HPA axis activity, disrupting normative neurodevelopment, and ultimately leading to the emergence of maladaptive behaviors (Nelson and Gabard-Durnam, 2020). It should be noted that the amount of stress needed to lower the stress reactivity threshold varies by individual due to certain genetic predispositions and history of *in utero* exposure to stress and other teratogens. Not all ELA paradigms immediately raise levels of corticosterone during the SHRP, but instead increase stress-sensitivity in adulthood (Lajud et al., 2012). For example, prenatal alcohol exposure is highly comorbid with ELA exposure in early childhood (up to 70%) and has been shown to increase the risk of developing maladaptive behaviors later in life due to the synergistic effects of the prenatal and early-life stressors on the HPA axis regulation in early childhood (Kambeitz et al., 2019; Lebel et al., 2019). In such cases, teratogenic exposure likely reduces the SHRP threshold or prevents the onset of the SHRP during perinatal development which increases the vulnerability of the brain to the damaging effects of ELA on early circuit development and glia proliferation. Finally, ELA resulting from malnutrition or poverty indirectly influences neurodevelopment by affecting overall growth and peripheral organ development which has been linked to neurodevelopmental delays (Nelson and Gabard-Durnam, 2020).

Glia proliferation, differentiation, and function are highly vulnerable to changes in HPA axis activity during development. However, a comprehensive discussion on how early HPA axis dysregulation affects astrocyte, microglia, and oligodendrocyte function is lacking. In this review, we explore the synergistic and overlapping effects of altered HPA axis development on glial function and neuron-glial communication which are imperative for synaptic pruning and myelination. Specifically, we theorize that ELA-induced stress often lowers the threshold of stress reactivity in early life leading to irregular HPA axis activity and an accumulation of glucocorticoids. Consequently, this alters neuronal excitability and synaptogenesis and induces lasting changes to glia production and function. In turn, these changes are detrimental to brain circuit refinement and optimization in the juvenile period. A detailed review of these underlying neurobiological mechanisms is critical for establishing ELA as a risk factor for psychiatric disorder development and will highlight specific targets for intervention.

Furthermore, ELA-induced HPA axis dysregulation causes unique changes to hippocampal (HPC) and prefrontal cortex (PFC) development which contribute to the emergence of specific behavioral phenotypes (Woolley et al., 1990; Nguyen et al., 2015; Guan et al., 2020). The effect of stress-induced extracellular glucocorticoid accumulation on neuronal excitability varies by brain region in development. Glucocorticoid-activated upregulation of neuronal excitability during the brain growth spurt drives the production of redundant synapses and alters glial number and function in HPC (Paolicelli et al., 2011; Schafer et al., 2012). Elevated levels of extracellular glutamate may be maintained into the juvenile period which delays or prevents effective synaptic pruning and disrupts the myelination of refined circuits (Parellada and Gassó, 2021). In contrast, elevated glutamatergic exposure represses glutamatergic and GABAergic neuronal excitability in the PFC leading to alterations in cortical disinhibition (Green et al., 2010; Ghosal et al., 2017). Due to the reciprocal connections of PFC with the hypothalamus and other subcortical regions including HPC and thalamus, alterations to PFC signaling lead to reduced inhibitory control over the HPA axis (Diorio et al., 1993; Herman, 2020). Consequently, a feed-forward loop is initiated wherein glucocorticoid production is further potentiated and this impairs synaptic pruning and myelination. We postulate that ELA contributes to an individual’s propensity towards either resilient or maladaptive behaviors by disrupting the delicate balance of synaptic pruning and axonal myelination which is necessary for adult brain circuitry refinement in and between these brain regions. Collective analysis of preclinical and clinical literature suggests targets for effective intervention to promote adaptive and resilient behaviors.

## Brain Circuits Are Developed During Perinatal Critical Periods

Critical periods are windows of heightened neuroplasticity during which the lack of certain stimuli or experiences has a dramatic, and often irreversible, effect on the development and function of the brain. They are characterized by experience-expectant processes that control the naissance and adequate maturation of brain circuits necessary for vision, hearing, language, and social and emotional development during infancy and early childhood (Nelson and Gabard-Durnam, 2020). Indeed, Nobel-prize winning studies conducted by Drs. David Hubel and Torsten Wiesel were some of the first to demonstrate that the absence of visual stimuli during a perinatal critical period results in lifelong visual impairments (Hubel and Wiesel, 1962). Following the closure of a critical period, the affected brain circuits become more resistant to future neuroplastic alteration. Biological processes occurring during early critical periods include cellular proliferation and differentiation, synaptogenesis, and the onset of myelination. As such, critical periods occur in infancy and early childhood. The specific length of critical periods depends on the rate of development of the putative circuit and varies in length from weeks to months (reviewed in Hensch, 2004).

### Key Neurodevelopmental Processes Characterizing Early Critical Periods

**Figure 1:**
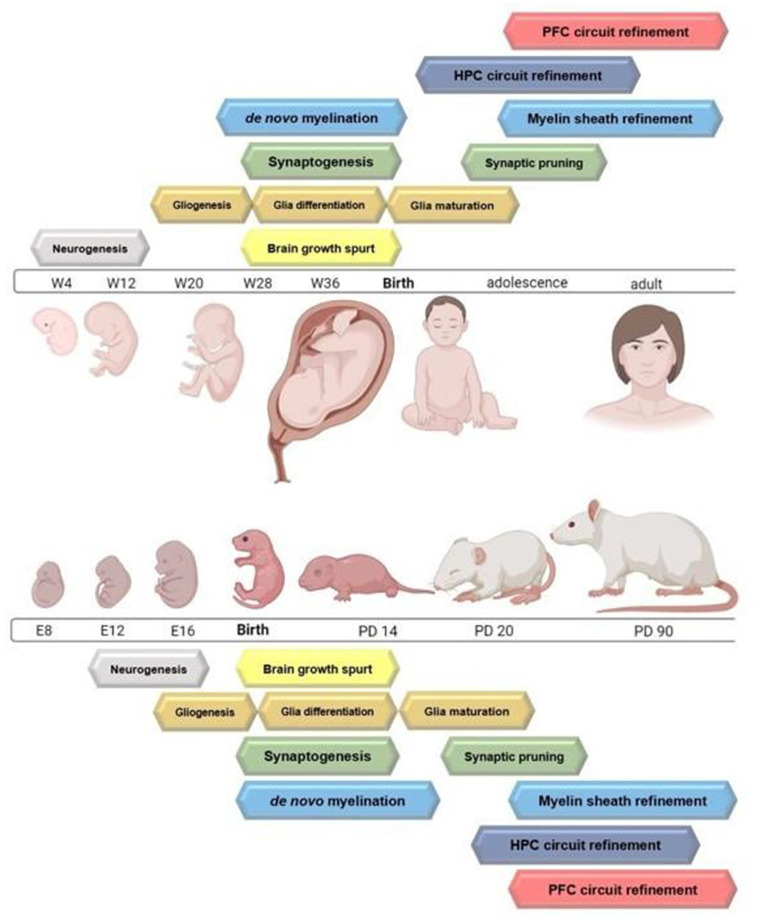
Timelines highlighting the peak of major neurodevelopmental processes occurring from gestation through adulthood in the rodent and humans. Circuit refinement of the hippocampus compared to the prefrontal cortex is also depicted. Figure created with BioRender.com.

Excluding microglia, all cells of the central nervous system (CNS) are derived from neuroepithelial cells, which are a type of multipotent neural stem cell (Williams and Price, 1995). Cellular proliferation in the mammalian CNS follows a sequential order, with the induction of neurogenesis coinciding with the formation of the ventricular zone (VZ). In rodents, the induction of neurogenesis begins on embryonic day (ED) 12, followed by gliogenesis which begins on ED 14 and peaks during the first 2 weeks of life (Levison et al., 1993; Zerlin et al., 1995; Lee et al., 2000). The primary location of cell proliferation in the CNS transitions from the VZ to the subventricular zone (SVZ) between ED 17 and 19, and is the dominant originating site for neuronal and glial progenitors later in gestation and throughout life (Sauvageot and Stiles, 2002). Analogous events occur in human neurodevelopment and are described in comparison to the rat in Figure 1. As the mammalian brain develops from the inside-out (building cortical layers above subcortical structures) radial glia replace neuroepithelial cells as the major progenitors for neurons and glia in the mammalian cortex. Disruption of cellular genesis in the CNS has lasting effects on neurodevelopment and CNS function.

Following cell proliferation, cell differentiation is driven by intrinsic and extrinsic cues occurring during highly regulated phases of gestation and early postnatal development. Growth factors provide important extracellular cues for the specific differentiation of astrocytes and oligodendrocytes during gestation and early postnatal life (for reviews see: Cameron et al., 1998; Sommer, 2006). Ciliary neurotrophic factor (CNTF) and leukemia inhibitory factor (LIF) initiate differentiation of the neural stem cells into astrocytes (Johe et al., 1996; Rajan and McKay, 1998). Whereas the signaling molecules sonic hedgehog (Shh), Notch, and Bone Morphogenic Proteins are essential for brain patterning (i.e., ventralization) and for expression of basic helix-loop-helix factors Olig1 and 2 which are required for oligodendrocyte differentiation (Lu et al., 2000; Zhou et al., 2000; Nery et al., 2001; Wang and Almazan, 2016). Secretion of platelet-derived growth factor (PDGF) by astrocytes triggers differentiation of oligodendrocyte precursor cells to oligodendrocytes when simultaneous with neuronal firing (Johe et al., 1996; Williams et al., 1997). Unlike astrocytes and oligodendrocytes, microglia are derived from yolk sac macrophages that invade the CNS prior to blood-brain barrier formation and subsequently mature into neuroglia (Lawson et al., 1990; Ginhoux and Prinz, 2015). Microglia secrete important growth factors and cytokines such as brain-derived neurotrophic factor and interleukin I that are essential for supporting neuro- and synaptogenesis, respectively (Giulian et al., 1988a, b; Parkhurst et al., 2013). Additionally, epigenetic modification of certain genes guides the differentiation of neural stem cells to functional glial cells in late gestation (for review see: Murao et al., 2016). For example, increased repression of histone marker *H3K27me3* in the promoter region of *Neurog1* and demethylation of Glial fibrillary acidic protein *(Gfap)* in neural stem cells induce the onset of astrogenesis (Hirabayashi and Gotoh, 2010; Murao et al., 2016). Similarly, removal of acetyl groups from histone proteins signals the onset for OPC differentiation to oligodendrocytes (Shen et al., 2005; Ye et al., 2009; Conway et al., 2012). Regulated cell genesis and differentiation prepare the neonatal brain for circuit development.

Following neuro- and gliogenesis, synaptogenesis is initiated, and early brain circuits are formed. In the mammalian brain, a massive wave of synaptogenesis occurs during the brain growth spurt (Dobbing and Sands, 1979). Glia are essential for modulating synaptogenesis during critical periods. Once an immature synapse is formed, astrocytic processes envelope the synaptic cleft to create the tripartite synapse which supports effective neurotransmission during synapse maturation (Chung et al., 2015; Farhy-Tselnicker and Allen, 2018). Further, astrocytes secrete cholesterol to stimulate the production of spontaneous excitatory postsynaptic potentials (EPSCs) and strengthen immature synapses (Mauch et al., 2001; Nägler et al., 2001; Allen and Barres, 2009; Chung et al., 2015; Reemst et al., 2016; Bosworth and Allen, 2017). A single astrocyte can maintain up to 140,000 synapses (Bushong et al., 2004). It should be noted that an overabundance of synaptic connections are created during this period (e.g., Bourgeois et al., 1994; Huttenlocher and Dabholkar, 1997; Stiles and Jernigan, 2010). Redundant synapses are phagocytosed during the phase of synaptic pruning and putative circuits remain (Stiles and Jernigan, 2010; Faust et al., 2021). Oligodendrocytes are involved in synaptogenesis, but the direct mechanisms remain unknown (Allen and Barres, 2009). However, once synapses are formed, oligodendrocytes are primarily responsible for insulating axons to support the survival and efficiency of relevant neural connections (Simons and Nave, 2015). Astrocytes, oligodendrocytes, and microglia are thus integral for proper synaptogenesis in early circuit development.

### Hippocampal and Prefrontal Cortex Development

The development of the hippocampus (HPC) and prefrontal cortex (PFC) is particularly, but not exclusively, vulnerable to environmental experience in early life (Kempermann and Gage, 1999; Raineki et al., 2019). The differential development of these two structures contributes to the unique effects that ELA-induced upregulations to excitatory signaling have on brain circuit development and function. For example, cell proliferation peaks during the brain growth spurt in the HPC and is maintained at a slower rate until the beginning of adolescence whereas the peak in cell proliferation occurs later in the PFC (Dobbing and Sands, 1973). Additionally, HPC granule cells in the dentate gyrus (DG) continue to proliferate throughout the lifespan (Nakafuku and del Águila, 2020). The HPC is composed of distinct sub-regions including the CA1, CA2, CA3, and DG, that are easily identifiable by different morphological traits and organization of pyramidal neurons (Khalaf-Nazzal and Francis, 2013). Moreover, 10–15% of the HPC is made up of GABAergic interneurons (Pelkey et al., 2017). During adolescence, HPC connections are fine-tuned and strengthened (Afroz et al., 2016). Leading up to and past adolescence, white matter tracts connecting HPC and PFC become more heavily myelinated. Particularly, white matter tracts connecting HPC to PFC are responsible for behaviors relating to inhibition, executive function, and attention and undergo extensive myelination in adolescence (Lebel and Beaulieu, 2011). Thus, HPC development is complex and environmental changes leading to increased glucocorticoid exposure have deleterious effects on HPC organization and connectivity.

Unlike in the HPC and other subcortical structures, the PFC continues to develop past adolescence into adulthood (Anderson et al., 2001; Koss et al., 2014). Composed of six sub-layers, the human PFC undergoes a period of increased volume and dendritic branching from early life until the onset of adolescence, before decreasing steadily until early adulthood during the phase of synaptic pruning (Koss et al., 2014). Indeed, rodent studies have shown that excitatory pyramidal neurons in the PFC experience a drastic decrease of both dendritic spines and excitatory synapses during the adolescent period (Koss et al., 2014; Drzewiecki et al., 2016; Drzewiecki and Juraska, 2020). Despite the pattern of decreasing dendritic spines and synapses, glial cell populations rise in the PFC at the onset of puberty indicating their increased role in adolescent circuit refinement involving PFC (Drzewiecki and Juraska, 2020). The PFC receives afferent projections from many structures in the brain, including the HPC, and PFC-temporal circuits that are specifically important for complex cognitive tasks that mature both during and after adolescence (Lebel and Beaulieu, 2011). Additionally, adolescence marks a time when neurons in the PFC are reorganized according to synaptic transmission. The maturation of GABAergic PFC interneurons is imperative for maintaining proper inhibitory control of excitatory PFC signaling (Caballero and Tseng, 2016; Drzewiecki and Juraska, 2020). Further, the development of these inhibitory neurons in the PFC is highly responsible for maintaining the proper functioning of excitatory projections that synapse onto behavior-related structures elsewhere in the brain. As the PFC is one of the last structures to develop, it is also susceptible to the effects of environmental experiences, such as ELA, on synaptic pruning and myelination.

## Brain Circuits Are Refined During Adolescent Sensitive Periods for Optimal Processing in Adulthood

Biomolecular processes occurring during perinatal critical periods lay the groundwork for optimizing neural circuitry in adolescence and refining brain circuitry in adulthood. Sensitive periods begin in late childhood and occur across a longer span of neurodevelopment. Experiences that occur during adolescent sensitive periods alter brain structure and function in an experience-dependent manner such that input is not required for, but may have a significant effect on, circuit refinement. Unlike critical periods, sensitive periods may be reopened to facilitate the refinement of brain circuitry that occurs as a result of synaptic pruning. However, this residual plasticity is limited (Knudsen, 2004). During this process, redundant synapses are removed from the brain and functionally-relevant connections are conserved and optimized through myelination to support adult cognition (Chechik et al., 1999; Knudsen, 2004). As such, the biological processes surrounding adolescent neurodevelopment necessitate proper glial function.

Nearly half of all CNS synapses created during the perinatal critical period are phagocytosed by microglia during the phase of synaptic pruning (Schafer et al., 2012; Zhan et al., 2014). Synaptic pruning results from the coordinated activity of all three major CNS glia. As aforementioned, microglia are the primary cells responsible for coordinating and performing synaptic pruning through the phagocytosis of synaptic material (Paolicelli et al., 2011) and the initiation of programmed cell death (Wakselman et al., 2008; Sominsky et al., 2018). Chemokine fractalkine signaling, *via* complement component cascade proteins and associated receptors, is a central mechanism by which microglia communicate with neurons to coordinate the removal of redundant synapses. During this process, excess synapses are tagged with *C1* and *C3* complement cascade proteins which bind to the *Cx3cr1* receptor on microglia to facilitate pruning of weak, surplus synapses. Synaptic pruning occurs earlier in PFC than HPC (Mallya et al., 2019). The repression of *Cx3cr1* receptor expression delays the onset of synaptic pruning and prevents microglia proliferation in the adolescent HPC. As a result, hyperconnectivity is observed in the HPC and consists of immature spines which form many weak synapses (Paolicelli and Gross, 2011; Zhan et al., 2014). An overabundance of circuit input from weak synapses disrupts neuronal synchrony, ultimately affecting functional connectivity of the HPC and PFC circuits (Pattwell et al., 2016; Honeycutt et al., 2020). Moreover, astrocytes play a pivotal role in coordinating synaptic pruning. Primarily, astrocytic processes monitor synapse strength and support excitatory signaling through glutamate recycling. However, astrocytes reserve the capacity to tag redundant synapses and directly phagocytose synapses (Stevens et al., 2007; Fair et al., 2008; Schafer et al., 2012; López-Murcia et al., 2015; Sultan et al., 2015; Bosworth and Allen, 2017). Reduced proliferation of astrocytes and/or disruption to astrocytic activity weakens mature synapses, prevents synapse maturation, and reduces synaptic pruning. Finally, oligodendrocyte precursor cells share the capacity to engulf synapses and aid in synaptic pruning (Buchanan et al., 2021). Interestingly, the proliferation, migration, and activity of OPCs bear a striking similarity to microglia.

The next phase of circuit refinement is axonal myelination, the process by which mature, myelinating oligodendrocyte (mOL) processes ensheath axons to support axonal survival and fine-tune axonal conduction velocity. The ontogenesis of mOLs in the mammalian brain is complex and varies by brain region and an organism’s age. Briefly, OPCs proliferate in three distinct gestational phases (Cui et al., 2012; Bergles and Richardson, 2016) and the differentiation of OPCs to mOLs occurs during the mammalian brain growth spurt; axons lengthen and myelination of functionally-relevant synapses is needed to support the emergence of early behaviors, such as suckling and vision. Much like neuro- and synaptogenesis, myelination begins in subcortical structures with HPC fiber tracts myelinated before cortical PFC axons. Myelination is arguably the longest neurodevelopmental process, with the fastest peak of myelination occurring in adolescence; this aligns with and follows the aforementioned process of synaptic pruning (Downes and Mullins, 2014; Kwon et al., 2020). One mOL may myelinate up to 50 axons in the CNS (Pfeiffer et al., 1993). Thus, abnormalities in OPC differentiation or mOL survival pose a major threat to axonal survival and circuit function. The onset and progression of these processes are critical for brain circuit optimization. Environmental experiences that alter adolescent myelination are known risk factors for the development of several psychiatric disorders including anxiety, schizophrenia, and depression (Makinodan et al., 2012; Forbes and Gallo, 2017).

All three major types of CNS glia work synergistically to facilitate myelination of axons with terminals serving functionally-relevant synapses. Following synaptic pruning in late childhood and early adolescence, microglia engulf viable OPCs as mediated by fractalkine signaling. Inadequate reduction of the OPC population prior to the onset of peak myelination in adolescence alters the OPC-mOL ratio, favoring mOLs, and leads to disruptions in typical axonal myelination patterns (i.e., mistargeted ensheathment of neuronal cell bodies and a thinning of the axonal myelin sheath). Thus, precise regulation of myelin content to axonal number is integral for proper myelination, and this balance is directly affected by microglial activity (Almeida et al., 2018; Nemes-Baran et al., 2020). Moreover, microglia are directly involved in myelin sheath pruning for refinement (Hughes and Appel, 2020) and microglia-OPC interactions mediate OPC differentiation to mOL (Wlodarczyk et al., 2017; Giera et al., 2018). Additionally, in an *in vitro* study, Pang et al. (2013) showed that astrocytes support long-term survival of OPCs, whereas microglia support oligodendrocyte differentiation and myelination. Astrocyte and oligodendrocyte communication has been shown to play an important role in myelin production. Astrocytes secrete platelet-derived growth factor (PDGF) which supports OPC proliferation and also inhibits immediate OPC differentiation (Raff et al., 1988; Richardson et al., 1988; McKinnon et al., 2005; Traiffort et al., 2020). Brain-derived neurotrophic factor (*Bdnf*) is also secreted by astrocytes, and *Bdnf* supports myelination during the brain growth spurt (Fletcher et al., 2018; Traiffort et al., 2020). Furthermore, astrocytes control the concentrations of Sonic hedgehog (*Shh*) produced which has a direct effect on OPC production throughout the forebrain (Traiffort et al., 2016).

Importantly, cellular and synaptogenesis occur during the SHRP, a perinatal neuroprotective period wherein HPA axis activation, and subsequently, glucocorticoid reactivity is significantly reduced (Schmidt et al., 2005; Slattery and Neumann, 2008). Alterations to neuronal physiology are harmful to cellular proliferation and synaptogenesis, and glia are most vulnerable to stress-induced changes to glucocorticoid exposure and glutamatergic signaling (Jauregui-Huerta et al., 2010). Glia express stress hormone and/or glutamate receptors and are thus directly impacted by the HPA axis and neuronal hyperactivity. For example, astrocytes replenish excitatory neurons with glutamate through glutamate recycling (Pfrieger and Barres, 1996; Sonnewald et al., 1997; Bélanger et al., 2011). Therefore, stress-induced changes to neuronal excitability directly affect astrocytic activity. Similarly, OPCs increase voltage-gated sodium channel expression in response to elevated glutamatergic signaling, which upregulates OPC-neuron communication and stimulates precocial OPC differentiation (Cheli et al., 2015). Moreover, mOL have a high bioenergetic consumption and are highly susceptible to glutamate-induced oxidative stress which can lead to apoptosis (Rosko et al., 2019; Meyer and Rinholm, 2021; Nakamura et al., 2021). Prolonged, unpredictable periods of ELA exposure are known to overwhelm the HPA axis, leading to a lowering of the stress threshold. Significant challenges to this threshold lead to lasting changes to glia proliferation and function and are correlated with altered brain connectivity and the emergence of maladaptive behaviors later in life. It should be noted that shorter, predictable periods of ELA prime the brain to experience future stress, and ultimately lead to the emergence of resilient and adaptive behaviors. In effect, increases to stress reactivity during the SHRP are quickly recovered.

## Early-Life Adversity (ELA) Alters The Proliferation and Function of Glia During The Perinatal Period and Leads to Dysregulated Synaptic Pruning and Myelination During Adolescence

### ELA Perturbs Synaptic Pruning in Adolescence *via* Immediate Upregulation of Glial Proliferation and Sustained Glial Dysregulation in HPC and PFC Leading to the Development of Inefficient Circuitry and Maladaptive Behaviors

**Figure 2:**
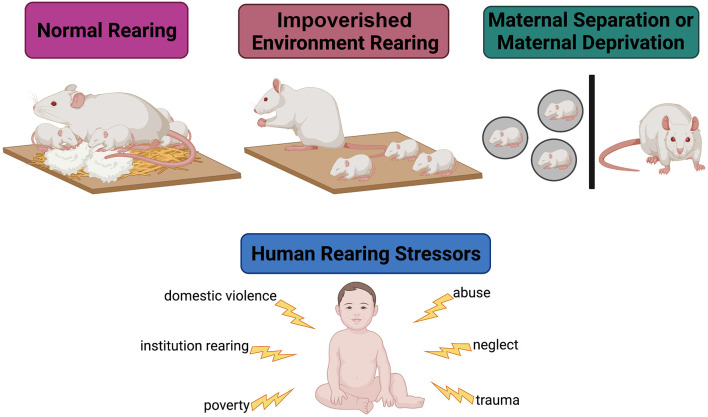
Modeling early-life adversity in the laboratory. Under normal rearing conditions, a rodent dam is provided with ample bedding and nesting material. In this environment, she engages in many nurturing behaviors (arched-back nursing, nest hovering, and pup licking) and a few aversive behaviors (stepping on, dragging, dropping, and actively avoiding) towards the pups. Under impoverished conditions, the dam is not provided with enough bedding or nesting material to create a nest. This environment causes stress in the dam, leading her to engage in less nurturing behaviors and more aversive behaviors towards the pups. In maternal separation models, the pups are removed from the dam to simulate neglect. These models are utilized to emulate human-experienced early-life stressors.

Exposure to ELA is associated with aberrant HPA axis activity, consequently disrupting the SHRP and leading to immediate changes in glial proliferation, synaptogenesis, and early circuit formation. Figure 2 illustrates the most common approaches to model ELA in rodents and summarizes known powerful ELA effectors in humans. Aberrant HPA axis activity leads to a prolonged and unpredictable increase in glucocorticoid circulation that increases neuronal excitability, directly and indirectly affecting glial proliferation and function (Slattery and Neumann, 2008). The varying effects of heightened glucocorticoid exposure on neuronal excitability depend on cell type (excitatory vs. inhibitory neuron), brain region, and age of the organism (neonatal vs. juvenile/adult) as glia receptor expression becomes more heterogeneous with age (Spitzer et al., 2019) and GABAergic neuronal activity is increased. For example, ELA alters the intrinsic excitability of ventral HPC pyramidal neurons by upregulating the expression of voltage-gated sodium channels and increasing long-term potentiation following extracellular glucocorticoid saturation. Conversely, ELA suppresses the excitability of medial PFC pyramidal neurons by reducing the expression of inwardly rectifying potassium (GIRK) channels (Teissier et al., 2020). As a result, cortical disinhibition is impaired and behavioral inhibition and anxiety-related behaviors are increased in adulthood (Sachs et al., 2013; Delli Pizzi et al., 2016).

Changes to neuronal excitability and extracellular glucocorticoid levels disrupt glia proliferation, differentiation, and function which ultimately impair circuit development through synaptogenesis. ELA-induced increases in neuronal excitation induce the formation of *de novo* dendritic spines. However, increased glucocorticoid exposure and neuronal excitation have deleterious effects on glial proliferation and function which disrupts synapse maturation and, in extreme cases, prevents synapse survival. Upregulation of neuronal excitability in ventral HPC immediately reduces the proliferation of astrocytes and delays astrocyte maturation *via* GFAP expression (Field, 1955; Nguyen et al., 2015; Abbink et al., 2019; Réus et al., 2019). The remaining astrocytes have a diminished capacity to metabolize glutamine, impairing support for the tripartite synapse and hindering early circuit development. Following ELA exposure, a compensatory overproduction of astrocytes is observed in juvenile HPC and PFC which leads to the creation of an abundance of immature synapses (Kwak et al., 2009; Llorente et al., 2009). Indeed, an overabundance of redundant input within and between HPC and cortical circuits leads to circuit desynchrony and is ultimately associated with deficits in anxiety-, fear-, and memory-related behaviors later in life (Paolicelli et al., 2011; Johnson and Kaffman, 2018; Zetter et al., 2021).

Additionally, ELA-induced upregulations to excitatory signaling and glucocorticoid exposure disrupt the proliferation and function of microglia (Catale et al., 2020). Microglia express glucocorticoid receptors and ELA-induced HPA axis dysregulation triggers immediate local proliferation of these neuroimmune cells (Hinwood et al., 2012; Rossetti et al., 2016; Catale et al., 2020). However, ELA exposure has opposing effects on microglial activity in HPC and PFC during critical periods of neurodevelopment. Specifically, cytokine release by microglia is acutely upregulated in both brain regions and is correlated with an increase in microglia soma volume (Wang et al., 2020). Moreover, Wang and colleagues (2020) demonstrate that ELA exposure upregulates the function of *Jmjd3* on *H3K27me3z* in HPC and PFC *via* epigenetic modification which facilitates sustained cytokine release from microglia in adolescence. Conversely, ELA exposure reduces the phagocytic activity of microglia in certain brain regions due to a disruption in fractalkine signaling between neurons and glia in the neonatal brain (Field, 1955; Ling, 1982; Winkler et al., 2017). Teratogenic exposure has varying effects on microglia cell number and function in HPC due to resulting neuronal apoptosis that immediately triggers a heightened neuroimmune response (Boschen et al., 2016; Ruggiero et al., 2018).

In normative neurodevelopment, the majority of microglia are sustained in an active, phagocytic state between infancy and late childhood to facilitate brain circuit refinement during the phase of synaptic pruning. As the organism ages, microglia mature and exhibit a less active state during which phagocytic activity is reduced. This phenotypic alteration begins in adolescence during the phase of myelination. Delpech et al. (2016) show that the normative maturation of microglia between early and late adolescence (when microglial states transition from active to surveyor) is delayed in HPC as a result of ELA exposure. Contrary to the expected, delayed maturation of microglia leads to uncoordinated synaptic pruning, resulting in the conservation of redundant, immature synapses (Zetter et al., 2021). This change is mediated in part by epigenetic modification of *Creb1*, *Sp1*, and *RelA* genes which are responsible for cellular migration and differentiation. Additionally, astrocytes release ATP in response to elevated levels of glucocorticoids which sustains microglial activation into adolescence (Davalos et al., 2005; Delpech et al., 2016; Yang et al., 2016). Fractalkine signaling, as discussed above, is also altered by ELA as a result of immediate and lasting reductions to *Cx3cl1* lipopolysaccharide binding protein (LBP) expression (Schwarz et al., 2011; Han et al., 2019). Like complement cascade factors, LBP is thought to mark nonfunctional synapses for elimination during circuit refinement. Wei et al. (2012) demonstrated that ELA exposure during the onset of synaptic pruning increases corticosterone levels and reduces LBP mRNA and protein expression in the HPC. Data from similar studies support this finding and suggest that aberrant microglia function early in life is correlated with a delay in synaptic pruning of connections in HPC during late childhood which leads to the emergence of redundant, less effective circuitry in adolescence (Wei et al., 2012, 2015; Delpech et al., 2016; Reshetnikov et al., 2020; Zetter et al., 2021). However, the effect of ELA on the phagocytic activity of microglia is region-specific. Indeed, ELA-induced neuroimmune activation coupled with aberrant neuronal signaling triggers the onset of neuronal oxidative stress and increases the phagocytic activity of microglia in PFC which may contribute to the dysfunction of local inhibitory circuits (Brenhouse et al., 2018; Gonzalez-Pardo et al., 2020). Collectively, disruptions to synaptic pruning lead to increased anxiety-like behaviors and decreased performance on hippocampal-dependent tasks in adulthood (Wei et al., 2012). Consistent with this conclusion, Bath et al. (2016) noted that ELA exposure resulted in the premature reduction of cellular markers for proliferation and differentiation, upregulation of markers of synapticity, and the early arrival of late-developing inhibitory neurons to the HPC.

In summary, these data indicate that ELA upregulates microglial and astrocytic function resulting from elevated levels of glucocorticoid and cytokine exposure. The observed glial hyperactivation is sustained through adolescence such that functions typical of more mature glial cells have a delayed developmental onset. As a result, synaptic pruning is delayed and hyperconnectivity is sustained in HPC and PFC, ultimately leading to the emergence of maladaptive behaviors in the juvenile period. Moreover, alterations to glia function deter the progression of synaptic pruning in later adolescence, such that it is less effective.

### ELA Perturbs Adolescent Myelination *via* Glial Dysregulation in Hippocampus and Prefrontal Cortex and Leads to the Development of Ineffective Circuitry and Maladaptive Behaviors

Delaying the onset and/or preventing the progression of synaptic pruning deleteriously affects the myelination of circuits in adolescence. With the existence of a greater number of immature synapses, myelination is less likely to be induced and is often delayed. Once induced, axonal ensheathment is impaired due to a reduction in myelin basic protein production, a paucity of viable OPCs prepared for differentiation resulting from precocial differentiation of mOLs, and a thinning of the myelin sheath with fewer layers. Indeed, ELA exposure during critical periods is correlated with alterations to the trajectory of myelination in adolescent HPC and PFC.

**Figure 3:**
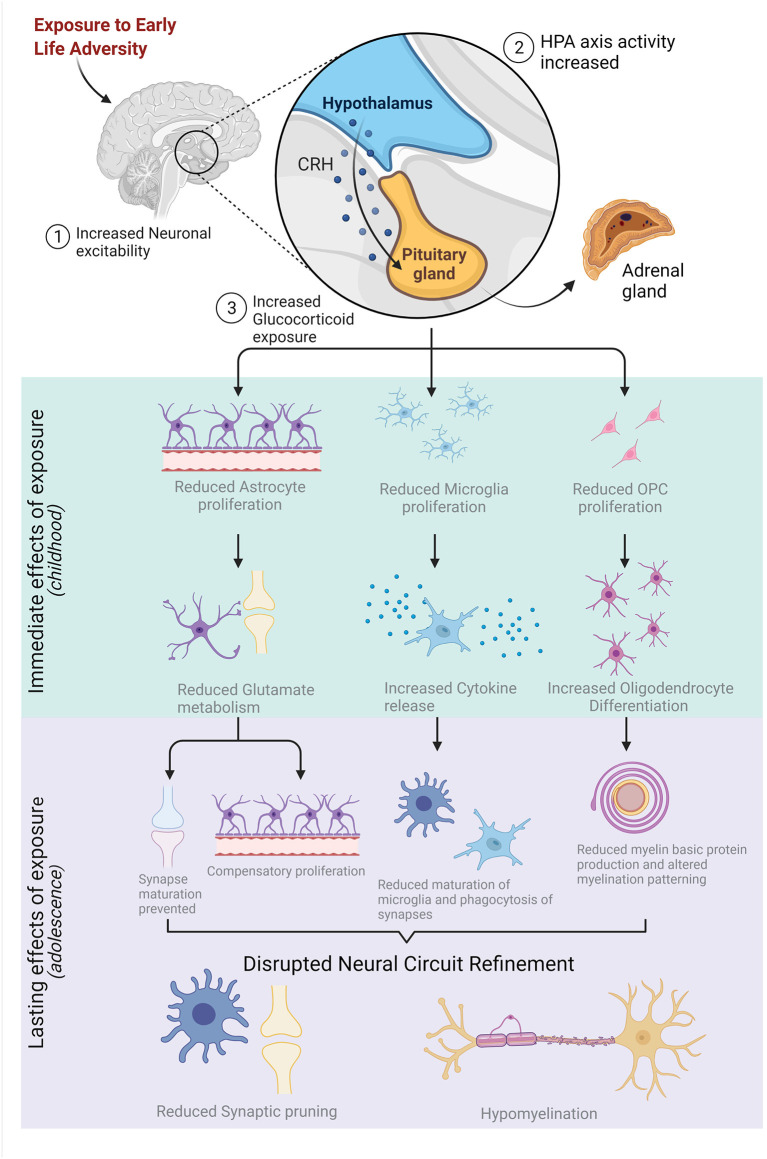
Early-life adversity (ELA) stimulates neuronal activity, increasing the activation of the hypothalamic-pituitary-adrenal (HPA) axis which leads to an over-production of glucocorticoids. Increased exposure to glucocorticoids immediately reduces the proliferation of astrocytes, microglia, and oligodendrocyte precursor cells (OPCs) which impacts the function and maturation of these glial cells. The lasting effects of ELA exposure on glia structure and function are highlighted in purple and, collectively, disrupt neural circuit refinement in adolescence by reducing synaptic pruning and resulting in hypomyelination. Figure created with BioRender.com.

In PFC and HPC, the increase in neuronal activity *via* glucocorticoid exposure drives the precocial differentiation of OPCs to myelinating oligodendrocytes (Kukley et al., 2010; Tanti et al., 2018; Treccani et al., 2021). Precocial differentiation of OPCs in the perinatal period reduces the population of available OPCs in the adolescent brain and leads to hypomyelination of salient white matter tracts in adult PFC and HPC (Teissier et al., 2020). The remaining OPCs, once differentiated to mOLs, produce insufficient levels of myelin basic protein due to the repression of myelin-related genes like MAG and MBP resulting from ELA (Kumar et al., 1989; Zeng et al., 2020). Additionally, ELA-induced upregulation of astrocytic activity indirectly impairs oligoglia activity. Glucocorticoid-induced alterations to neuronal excitability reduce GFAP expression in astrocytes which is correlated with reductions to white matter integrity in HPC and PFC and increased anxiety-like behaviors in adolescence (Zeng et al., 2020). Finally, disruptions to microglial activity alter the onset of myelination *via* phagocytosis of viable OPCs as mentioned previously (Wlodarczyk et al., 2017; Almeida et al., 2018; Giera et al., 2018; Nemes-Baran et al., 2020). Collectively, these findings indicate that ELA exposure leads to the downstream dysfunction of glia and delays the onset and progression of synaptic pruning, ultimately leading to deficits in effective myelination. Indeed, the emergence of many psychiatric disorders later in adolescence and young adulthood have been linked to ELA exposure during neurodevelopment through disruptions to the onset and progression of late-occurring neurodevelopmental processes (Fernandez and Garner, 2007; Coghlan et al., 2012; Bitanihirwe and Woo, 2014). These relationships are summarized in Figure 3.

## Alterations to The Neuroanatomical Organization of Brain Circuits in Adolescence Result in Varying Changes to Functional Connectivity Underlying Behavior in Adolescence and Adulthood

ELA reduces the volume of HPC and PFC in adolescence and adulthood. Specifically, as a result of normative synaptic pruning, cortical and subcortical gray matter (composed of cell bodies and axons) is thinned in adolescence (Gorka et al., 2014; Hanson et al., 2015; Underwood et al., 2019; Monninger et al., 2020). However, ELA disrupts this process and leads to alterations in PFC and HPC that do not follow the predetermined developmental trajectory of these regions (Sapolsky, 1996; Kim and Yoon, 1998; Gorka et al., 2014; Hanson et al., 2015; Calem et al., 2017). For example, many prominent hippocampal subregions (CA1, CA3, and the granule cell layer of the dentate gyrus) are smaller in children with ELA exposure (Teicher et al., 2012). PFC volume is similarly reduced following ELA exposure. We expect that ELA-related changes in HPC or PFC volume may be attributed to cellular apoptosis and/or hypomyelination as opposed to normative synaptic pruning. It should be noted that ELA exposure causes lateralization in the growth of these structures and is correlated with circulating cortisol levels (Dahmen et al., 2018).

ELA alters the connectivity between brain regions important for impulse control, emotional regulation, and memory (Miller and Cohen, 2001; Arnsten, 2009; Jin and Maren, 2015). As previously described and summarized in Figure 3, glial dysregulation prevents appropriate synaptic pruning and results in increased axonal connectivity between some structures. The discovery of increased connectivity was first observed between the PFC, HPC, and the amygdala of institutionalized children using magnetic resonance imaging and was correlated with disruptions in circuit function (Sullivan and Holman, 2010). Rearing of children in orphanages is a known risk factor for reaching appropriate neurodevelopmental milestones (Theoretical Empirical Practical Rationale, 2008; Hostinar et al., 2012) likely due to the reduction in personalized care and lack of routine in some homes. Specifically, formerly institutionalized children exhibited precocial amygdala-PFC connectivity, a pattern atypical of their developmental stage, which is correlated with an increase in anxiety-like behaviors and reduced inhibitory control (Gee et al., 2013; Silvers et al., 2016). Indeed, preclinical studies using rodent models of perinatal maternal separation indicate that amygdala-HPC and amygdala-PFC functional connectivity is increased in adolescence and is correlated with an upregulation in anxiety-like behaviors (Johnson and Kaffman, 2018). Histological assessment of brain tissue samples collected from these animals confirms that maternal separation stress leads to the precocial innervation of medial PFC from the basolateral amygdala in adolescence. Interestingly, the increased connectivity was observed earliest in the female adolescent brain (Honeycutt et al., 2020).

ELA-induced alterations to regional volume and circuit functional connectivity lead to the emergence of maladaptive behaviors (Roceri et al., 2004; Holmes and Wellman, 2009; van Harmelen et al., 2010; Moriguchi and Hiraki, 2013; Gorka et al., 2014; Van Harmelen et al., 2014a, b). Notable impairments to executive function are observed in preclinical (Lovic and Fleming, 2004; Roceri et al., 2004; Holmes and Wellman, 2009; Liston and Gan, 2011) and clinical studies (Moriguchi and Hiraki, 2013). Thus, ELA-exposed adolescents and adults exhibit impaired future planning, abstract conceptualization, and emotional gating (Hostinar et al., 2012; French and Carp, 2016). Moreover, alterations to HPC volume and connectivity are associated with increased risk for developing depression (Baaré et al., 2010; Rao et al., 2010) and stress-related disorders (Geuze et al., 2005; Kitayama et al., 2005; Smith, 2005; Anda et al., 2006; Etkin and Wager, 2007). Structural and functional alterations within the PFC and HPC as a consequence of ELA also affect communication between these structures, contributing to the emergence of maladaptive behaviors. Patients with neuropsychiatric and stress-related disorders showed altered functional connectivity in PFC-HPC circuitry which contributes to deficits in working memory (Genzel et al., 2015; Jin and Maren, 2015; Poletti et al., 2016; Calabro et al., 2020). Lambert et al. (2019) showed that children with ELA exposure exhibited HPC-dependent learning deficits and this impairment increases with age. Increased middle frontal gyrus and reduced intraparietal sulcus activation during the encoding phase of learning emerge with increasing age in children exposed to violence and may reflect the greater effort required to maintain attention due to less efficient short-term memory storage.

Furthermore, ELA exposure results in enhanced sensitivity to stress hormones *via* impairments to negative feedback loops which control stress reactivity in HPC and PFC (Maniam et al., 2014; Van Bodegom et al., 2017). Glucocorticoids are essential for cellular and behavioral responsiveness to stressful situations and bind to mineralocorticoid receptors (MRs) and glucocorticoid receptors (GRs) on neurons and glia. These two receptors have different affinities for glucocorticoids and their expression is brain-region specific. Increased expression of GRs with limited MR colocalization in the HPC, PFC, hypothalamus, and pituitary facilitates homeostatic regulation of the HPA axis *via* negative feedback loops (Reul and De Kloet, 1986; Sapolsky and Meaney, 1986; Fuxe et al., 1987; Van Eekelen et al., 1988; Meaney et al., 1996; Jankord and Herman, 2009). However, ELA leads to a lasting reduction in GR expression in the adolescent and adult HPC which plays a major role in alterations to functional connectivity and contributes to behavioral impairment associated with heightened stress reactivity (Champagne, 2013; Liu and Nusslock, 2018). As such, pharmaceutical intervention with a GR antagonist is not recommended. It should be noted that some amount of glucocorticoid exposure is necessary for the development of brain circuits underlying the neurobiological response to stress and fear and their regulation. Thus, blockade of GR in certain brain regions during the SHRP may be harmful to brain development and would have increasingly detrimental effects on neurodevelopment in organisms exposed to ELA due to the reduction in GR expression. Schmidt et al. (2005) demonstrated that blockade of glucocorticoid receptors during the SHRP led to increased basal corticosterone levels, suggesting an impairment in the self-regulation of the HPA axis following GR receptor antagonism. When administered during an ELA paradigm, GR antagonism had a synergistic effect on basal corticosterone levels during the SHRP—corticosterone levels were four times greater than in pups just exposed to the ELA paradigm. It may be predicted that antagonism of such receptors on glia cells, namely microglia, severely upregulates the neuroimmune system and is therefore disadvantageous. Pharmaceutical interventions providing partial antagonism or competitive inhibition may be more suitable solutions for preventing the lowering of the stress reactivity threshold during ELA exposure. Reductions to GR expression are associated with alterations to functional connectivity in adolescence (Arnett et al., 2015). More research is needed to identify how ELA alters GR expression on glia, specifically.

Finally, it has been argued that increased circuit connectivity might be adaptive and promote some resilient behaviors. For example, increased connectivity and reactivity to novel stimuli may provide institutionalized children with a better means of interacting with the unpredictable and stressful nature of their environments (Tottenham, 2012). Moreover, previously institutionalized children exhibit more effective aversive learning during a threat-discrimination task that may be beneficial for survival (Silvers et al., 2016). Increased activation of the amygdala, HPC, and PFC circuits in institutionalized children and adolescents likely contributes to greater assessment of potential threats in their environment, increasing their capacity for self-preservation. However, extreme hyperconnectivity is linked to the onset of neuropsychiatric disorders.

## Targeted Treatment Interventions for Ela Exposure: Preclinical and Clinical Findings

**Figure 4:**
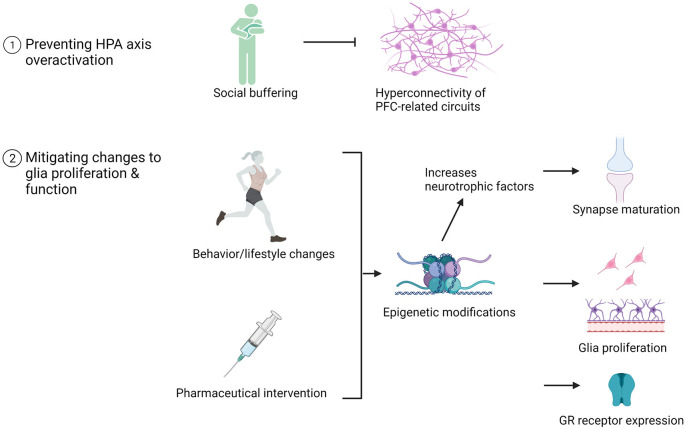
Examples of pharmaceutical and behavioral therapeutic interventions that promote neurodevelopment in adolescents with a history of ELA. The impact of these interventions on brain structure, function, and glia number are listed.

Environmental and pharmacological therapies mitigate ELA-induced structural and functional alterations to HPC and PFC in adolescence and adulthood. While human studies allow us to explore the effect of ELA on behavioral outcomes, epigenetic modifications, functional connectivity, and HPA axis function, animal models facilitate the investigation of the neurobiological underpinnings supporting these behavioral alterations. As a result, novel targets for intervention are discovered. We have highlighted several glia-centric mechanisms as targets for intervention: preventing HPA axis dysregulation during critical and sensitive periods of neurodevelopment, mitigating changes to glia proliferation, and restoring normative glia function in adolescence. These therapies are summarized in Figure 4 and described in more detail below.

### Preventing HPA Axis Dysregulation During Critical and Sensitive Periods of Neurodevelopment

Several behavioral interventions effectively regulate HPA axis activity in cases of ACE/ELA exposure. Increasing nurturing caregiving during or following ELA exposure prevents the emergence of maladaptive behaviors and corrects behaviors associated with anxiety and stress-related hyperreactivity. During ELA exposure, the increased presence of an active adult caregiver provides social buffering which prevents excessive activation of the infant or child HPA axis. Social buffering is most effective in childhood and works by securing a more predictable and rewarding rearing environment to compensate for a child’s prolonged exposure to unpredictable stress (Gunnar et al., 2015). Adoption is a form of social buffering for children previously in foster care. Relaxation of HPA axis activity resulting from social buffering supports PFC development wherein the hyperconnectivity between PFC and HPC or the amygdala is prevented. This is correlated with attenuating robust behavioral distress resulting from ELA (Gunnar et al., 2015; Barry et al., 2017). The Attachment and Biobehavioral Catch-up (ABC) intervention is an effective program wherein the caregiver-child relationship is ameliorated by teaching biological or foster parents the importance of being guided by a child’s needs early in life so as to construct an environment where the child can process emotions to stimulate self-regulation (Dozier et al., 2006). Children whose foster parents received the ABC intervention have HPA axis regulation comparable to non-institutionalized children and exhibit fewer behavioral problems (Dozier et al., 2006; DePasquale et al., 2018). Additionally, the Multidimensional Treatment Foster Care for Preschoolers (MTFC-P) program, developed by the Oregon Social Learning Center, is an intensive intervention that provides robust resources and support to both the caregiver and the child and has a similar regulatory effect on HPA axis activity (Fisher et al., 1999, 2011). Amelioration of the caregiver-child bond early in life is a strong intervention for correcting HPA axis dysregulation resulting from ELA exposure and can prevent downstream consequences on glia proliferation and function.

Nevertheless, providing caregiver support (including adoption) in late childhood and adolescence continues to benefit the child’s neurodevelopment and correct maladaptive behaviors resulting from ELA. Increasing nurturing caregiving during late neurodevelopment does not strongly affect PFC development, but is suggested to impact aspects of functional connectivity. Indeed, adolescent intervention *via* social buffering successfully attenuates abnormal threat responses but does not correct for learned fear which is less malleable in adolescence (Fisher et al., 2006; Gunnar et al., 2019; Robinson-Drummer et al., 2019).

### Mitigating Changes to Glia Proliferation and Restoring Normative Glia Function in Adolescence

ABC training has been shown to correct several ELA-induced modifications to the epigenome in support of synaptogenesis and myelination of mature neuronal connections (Hoye et al., 2020). Attachment training, designed to increase parental nurturing behaviors, is advantageous in correcting the maladaptive HPA axis response to stressful situations early in life. Findings from rodent research elucidate the epigenetic mechanism by which ELA continues to alter behavior in adulthood. For example, adult rats treated with a DNA methylation inhibitor showed recovery of ELA-induced aberrant methylation of the gene for Brain-Derived Neurotrophic Factor, *Bdnf* (Roth et al., 2009). Downregulating *Bdnf* gene expression *via* aberrant methylation during development can have detrimental effects on long-term potentiation and synaptic transmission which are vital for dendritic plasticity and cell survival during synaptogenesis (for review see: Lu et al., 2014). *Bdnf*, produced by microglia, supports synaptogenesis (Parkhurst et al., 2013) and facilitates astrocytic involvement in synapse maturation of putative circuits (Bergami et al., 2008). Indeed, preclinical research demonstrates that preventing alterations to *Bdnf* methylation through pharmacological (Doherty, 2019) intervention or increased caregiving prevented the development of certain behavioral deficits in adulthood (Keller et al., 2019).

Behavioral interventions beyond ameliorating the caregiver-infant relationship have a profound effect on mitigating ELA-induced alterations to circuit refinement in adolescence when studied in rodents. First, exposure to stimulating, complex environments induces glia proliferation which may combat the effect of ELA on the glia population (Voss et al., 2013; Tomlinson et al., 2016; Vivar and van Praag, 2017). Environmental complexity (EC) includes exposure to several stimulators of neuroplasticity including novel objects (i.e., changing toys in the cage) and social stimuli (i.e., group housing; van Praag et al., 2000; Moriceau et al., 2009; Kentner, 2015) which facilitate learning. It should be noted that in some cases increased maternal care is considered as a form of EC (Winkelmann-Duarte et al., 2011). Specifically, EC prevents HPA axis hyperactivity and promotes synaptogenesis and maturation of synapses in HPC and PFC (Monteiro et al., 2014; Boschen et al., 2017; Dandi et al., 2018; Smith et al., 2018). The beneficial effect of EC on HPC structure and function is highest when administered in adolescence and/or young adulthood and may be mediated through glia proliferation and function (for review see; Ohline and Abraham, 2018). For example, EC during young (~3 months of age) and older adulthood (~1 year of age) induces the differentiation of OPCs leading to increased myelination in HPC which facilitates neuronal signaling (Pusic and Kraig, 2014). Moreover, EC increases astrocyte density in the HPC (Winkelmann-Duarte et al., 2011), and reverses the immediate effect of ELA on astrocyte number, which may prevent reductions in glutamine metabolism which are damaging to the function of tripartite synapses. Collectively, these neurobiological alterations reverse cognitive impairments caused by ELA exposure including fear learning as well as depressive- and anxiety-like behaviors (do Prado et al., 2016; Dandi et al., 2018; Borba et al., 2021).

Increased voluntary aerobic activity is a potent stimulator of OPC proliferation and regulates the neuroimmune system. Due to the ongoing development of the PFC in adolescence, the timing of exercise intervention differentially affects OPC proliferation in the cortex compared to subcortical structures. Increased exercise in young adulthood compared to adolescence is most likely to generate OPCs in PFC (Tomlinson et al., 2018). However, there is increasing evidence that exercise induces OPC proliferation as well as neurogenesis in the adult HPC (Islam et al., 2020). The research on exercise interventions for children exposed to ELA is nonexistent, leaving a wide window for future research efforts. However, rodent studies provide evidence that aerobic exercise rescues alterations to HPC structure and cognitive capacity following teratogenic exposure (alcohol; Boschen et al., 2014). Further, voluntary wheel running has been shown to rescue alterations to microglia number and function in the adult cerebellum in a rodent model of Fetal Alcohol Spectrum Disorders (Gursky et al., 2020) and is predicted to support myelination in the adolescent brain of children with a history of prenatal alcohol exposure (Tomlinson et al., 2016; Wozniak et al., 2019). Exercise increases astrocytic coverage of blood vessels (Leardini-Tristaõ et al., 2020) and upregulates the number of GFAP-positive astrocytes in the HPC following ELA exposure (Belaya et al., 2020; Li et al., 2021). These astrocytic changes prevent neurodegeneration (Leardini-Tristaõ et al., 2020), cognitive impairment associated with Alzheimer’s disease (Belaya et al., 2020), depression, and lowered HPC volume (Li et al., 2021). Microglial activation and population in HPC are reduced following exercise (Kohman et al., 2013; Mee-inta et al., 2019). High-intensity exercise serves a general neuroprotective role by reducing microglial-associated inflammation (Zaychik et al., 2021), which is also associated with increases in *BDNF* expression and improvements in spatial working memory (Xiong et al., 2015). Together, these data highlight how exercise is expected to reverse some of the negative consequences of ELA on glia cell proliferation and function so as to ameliorate behavioral deficits.

A “super-intervention” which includes increased aerobic activity followed by EC exposure mitigates the impact of ELA on circuit development and refinement (Hamilton et al., 2012, 2014). The application of this intervention during adolescence and adulthood in rodent models of Fetal Alcohol Spectrum Disorders promotes adult neurogenesis in the HPC and supports the integration of these immature neurons into existing circuits (Hamilton et al., 2012, 2014). Though little is known about the impact of the “super-intervention” on glia number and function, it is predicted that this intervention may strengthen existing neural connections between HPC and PFC, possibly *via* changes to myelination. Further, it was also shown that this intervention rescues hippocampal learning deficits in alcohol-exposed animals (Hamilton et al., 2014). Though outside of the traditional ELA context, these data support the claim that environmental and behavioral interventions positively support neurodevelopment in adolescence.

Finally, pharmaceuticals known to alter the epigenome may one day be suitable for application for preventing the impact of ELA on brain circuit refinement. As previously discussed, ELA-induced epigenetic modifications which cause reductions to GR expression in HPC and PFC alter the functional connectivity between these structures and lead to the emergence of working memory- and anxiety-related deficits (Arnett et al., 2015). Weaver et al. (2005) have demonstrated that treatment with the amino acid L-methionine, a methyl group donor, in adulthood normalized methylation patterns of the *GR* gene and normalized HPA axis activity in rats that experienced low levels of maternal nurturing behaviors. GRs are widely expressed in microglia, oligodendrocytes, and astrocytes (Vardimon et al., 1999; Melliar-Smith and Moser, 2015). Microglial function and activation are regulated in part by glucocorticoid (GC) binding (Melliar-Smith and Moser, 2015). GCs regulate the maturation and myelinating activity of OLs, however, direct involvement of GRs is lacking. GCs also influence astroglial activity and cell number (Mee-inta et al., 2019; Zeng et al., 2020). We can infer that, based on the literature discussed above, ELA increases circulating GCs and that the saturation of GCs leads to downregulation of GR expression on glial cells. Ultimately, a reduction in GR number on these cells may cause functional changes and influence their survival. It is unknown how supplementation with methyl donors such as L-methionine might influence synaptic pruning and myelination in adolescents with a history of ELA. Microglia and astrocytes also express histone deacetylase (HDAC) enzymes, which are proteins that prevent transcription of genes and whose activity can be influenced by HDAC inhibitors (HDACi: Weaver et al., 2004; Volmar and Wahlestedt, 2015). In cell culture, microglia and astrocytes treated with an HDACi (SAHA or ITF2357) showed increased histone acetylation—an important cellular marker for gene transcription and OPC differentiation (Faraco et al., 2009). In a mouse model of maternal separation, treatment with the HDACi, sodium valproate, increased global histone 3 acetylation in the whole brain of pups, and marginally increased the maternal behaviors (i.e., licking/nursing and pup investigation) of this generation in adulthood (Burenkova et al., 2019). Zebularine—a DNA methyltransferase inhibitor—has also been successful at decreasing methylation, and subsequently increasing gene expression, of *Bdnf* in adult rats who experienced ELA (Roth et al., 2009). HDACi drugs or other epigenetic drugs may mitigate behavioral consequences of ELA.

## Discussion and Clinical Relevance

Aberrant glial proliferation and function resulting from exposure to ELA delays the onset of synaptic pruning in adolescence, with consequential precocial formation of redundant, immature synapses. This feed-forward loop of impaired adolescent circuit refinement results in the incomplete myelination of an abundance of weak synapses, ultimately leading to the emergence of maladaptive behaviors. Indeed, dysregulated synaptic pruning and myelination in adolescence is correlated with changes in behavior and cognitive capacity. In most cases, ELA exposure leads to a prolonged elevation in HPA axis activity during critical periods of neurodevelopment. Immediate changes to glia proliferation and function ultimately cause downstream impairments to glia function during later adolescent sensitive periods of neurodevelopment.

ELA-induced alterations to neural circuits are region-specific as the development of certain structures is not uniform across gestation and early childhood (Dobbing and Sands, 1973). We describe how the disruption of circuit refinement at the cellular level contributes to aberrant functional connectivity between and within the HPC and PFC. Our review of the literature has uncovered that ELA leads to hyperconnectivity of HPC and PFC-dependent circuits in adolescence and young adulthood due to reduced synaptic pruning. Consequently, the abundance of weak, immature synapses produces a disorganized landscape for myelination. Moreover, ELA exposure prevents the production of myelin basic protein in the adolescent brain, reducing the thickness of existing myelin sheaths. As a result, functional connectivity within and between HPC and PFC is impaired resulting in the emergence of maladaptive behaviors. These include increased anxiety-like behaviors and reduced capacity for impulse control, emotional regulation, and memory encoding.

Our review of glia-derived mechanisms underlying impairments to circuit refinement in adolescence following ELA exposure highlights several targets for effective therapeutic intervention. Moreover, behavioral interventions, which are accessible and affordable, strongly benefit structural and functional repair of HPC and PFC by preventing hyperactivation of the HPA axis in children and upregulating myelination in adolescents exposed to ELA. These include therapies which strengthen the caregiver-child bond such as the ABC intervention (Dozier et al., 2006) and the MTFC-P program (Fisher et al., 1999). At the neurobiological level, these interventions interact with the genome and increase the expression of growth factors which support normative glial function. Additionally, exposure to a complex environment or increased voluntary aerobic exercise are predicted to have a significant effect on mitigating the reduction in glia proliferation and maturation resulting from ELA. Future research would benefit from validating that these behavioral manipulations affect synaptic pruning or myelination in the ELA-exposed adolescent brain. Continued work under this lens will help us better understand preventative measures and interventions to improve the quality of life for children and families overall.

## Author Contributions

All authors collaboratively conceived the focus of this review and contributed to the draft and final versions of the manuscript. KM, NC, TR, and AK edited the manuscript and interpreted the collective findings of the preclinical and clinical research to understand the immediate and lasting effects of early-life adversity on glia-driven processes in neurodevelopment. KM, TC, and SK conducted the majority of the literature review and created the four included figures. All authors contributed to the article and approved the submitted version.

## Conflict of Interest

The authors declare that the research was conducted in the absence of any commercial or financial relationships that could be construed as a potential conflict of interest.

## Publisher’s Note

All claims expressed in this article are solely those of the authors and do not necessarily represent those of their affiliated organizations, or those of the publisher, the editors and the reviewers. Any product that may be evaluated in this article, or claim that may be made by its manufacturer, is not guaranteed or endorsed by the publisher.

## Abbreviations

ABC: Attachment and Biobehavioral Catch-up program
ACE: Adverse childhood experience
BDNF: Brain derived neurotrophic factor
CNTF: Ciliary neurotrophic factor
DG: Dentate Gyrus
EC: Environmental complexity
ED: Embryonic day
ELA: Early-life adversity
EPSC: Excitatory postsynaptic potential
GC: Glucocorticoid
GFAP: Glial fibrillary acidic protein
GR: Glucocorticoid receptor
HDAC: Histone deacetylase
HDACi: Histone deacetylase inhibitor
HPA: Hypothalamic-pituitary-adrenal axis
HPC: Hippocampus
LBP: Lipopolysaccharide binding protein
LIF: Leukemia inhibitory factor
MBP: Myelin basic protein
mOL: Myelinating oligodendrocyte
MR: Mineralocorticoid receptor
MTFC-P: Multidimensional Treatment Foster Care for Preschoolers
OL: Oligodendryocyte
OPC: Oligodendrocyte precursor cell
PDGF: Platelet-derived growth factor
PFC: Prefrontal cortex
SHH: Sonic Hedgehog
SHRP: Stress hyporesponsive period
SVZ: Subventricular zone
VZ: Ventricular zone.

## References

[B1] Abbink M. R. van Deijk A. L. F. Heine V. M. Verheijen M. H. Korosi A. (2019). The involvement of astrocytes in early-life adversity induced programming of the brain. Glia 67, 1637–1653. 10.1002/glia.23625 31038797 PMC6767561

[B2] Afroz S. Parato J. Shen H. Smith S. S. (2016). Synaptic pruning in the female hippocampus is triggered at puberty by extrasynaptic GABAA receptors on dendritic spines. eLife 5:e15106. 10.7554/eLife.15106 27136678 PMC4871702

[B4] Allen N. J. Barres B. A. (2009). Neuroscience: Glia—more than just brain glue. Nature 457, 675–677. 10.1038/457675a 19194443

[B5] Almeida R. G. Pan S. Cole K. L. H. Williamson J. M. Early J. J. Czopka T.. (2018). Myelination of neuronal cell bodies when myelin supply exceeds axonal demand. Curr. Biol. 28, 1296–1305.e5. 10.1016/j.cub.2018.02.068 29628374 PMC5912901

[B6] Anda R. F. Felitti V. J. Bremner J. D. Walker J. D. Whitfield C. Perry B. D.. (2006). The enduring effects of abuse and related adverse experiences in childhood: a convergence of evidence from neurobiology and epidemiology. Eur. Arch. Psychiatry Clin. Neurosci. 256, 174–186. 10.1007/s00406-005-0624-4 16311898 PMC3232061

[B7] Anderson V. A. Anderson P. Northam E. Jacobs R. Catroppa C. (2001). Development of executive functions through late childhood and adolescence in an Australian sample. Dev. Neuropsychol. 20, 385–406. 10.1207/S15326942DN2001_5 11827095

[B8] Arnett M. G. Pan M. S. Doak W. Cyr P. E. P. Muglia L. M. Muglia L. J. (2015). The role of glucocorticoid receptor-dependent activity in the amygdala central nucleus and reversibility of early-life stress programmed behavior. Transl. Psychiatry 5:e542. 10.1038/tp.2015.35 25849981 PMC4462600

[B9] Arnsten A. F. T. (2009). Stress signalling pathways that impair prefrontal cortex structure and function. Nat. Rev. Neurosci. 10, 410–422. 10.1038/nrn2648 19455173 PMC2907136

[B10] Baaré W. F. C. Vinberg M. Knudsen G. M. Paulson O. B. Langkilde A. R. Jernigan T. L.. (2010). Hippocampal volume changes in healthy subjects at risk of unipolar depression. J. Psychiatr. Res. 44, 655–662. 10.1016/j.jpsychires.2009.12.009 20096419

[B11] Barry T. J. Murray L. Fearon P. Moutsiana C. Johnstone T. Halligan S. L. (2017). Amygdala volume and hypothalamic-pituitary-adrenal axis reactivity to social stress. Psychoneuroendocrinology 85, 96–99. 10.1016/j.psyneuen.2017.07.487 28843903 PMC5632999

[B12] Bath K. G. Manzano-Nieves G. Goodwill H. (2016). Early life stress accelerates behavioral and neural maturation of the hippocampus in male mice. Horm. Behav. 82, 64–71. 10.1016/j.yhbeh.2016.04.010 27155103 PMC5308418

[B13] Bélanger M. Allaman I. Magistretti P. J. (2011). Brain energy metabolism: focus on Astrocyte-neuron metabolic cooperation. Cell Metab. 14, 724–738. 10.1016/j.cmet.2011.08.016 22152301

[B14] Belaya I. Ivanova M. Sorvari A. Ilicic M. Loppi S. Koivisto H.. (2020). Astrocyte remodeling in the beneficial effects of long-term voluntary exercise in Alzheimer’s disease. J. Neuroinflammation 17:271. 10.1186/s12974-020-01935-w 32933545 PMC7493971

[B15] Bergami M. Santi S. Formaggio E. Cagnoli C. Verderio C. Blum R.. (2008). Uptake and recycling of pro-BDNF for transmitter-induced secretion by cortical astrocytes. J. Cell Biol. 183, 213–221. 10.1083/jcb.200806137 18852301 PMC2568011

[B16] Bergles D. E. Richardson W. D. (2016). Oligodendrocyte development and plasticity. Cold Spring Harb. Perspect. Biol. 8:a020453. 10.1101/cshperspect.a020453 PMC4743079 26492571

[B17] Bitanihirwe B. K. Y. Woo T. U. W. (2014). Perineuronal nets and schizophrenia: the importance of neuronal coatings. Neurosci. Biobehav. Rev. 45, 85–99. 10.1016/j.neubiorev.2014.03.018 24709070 PMC4447499

[B18] Borba L. A. Broseghini L. D. R. Manosso L. M. De Moura A. B. Botelho M. E. M. Arent C. O.. (2021). Environmental enrichment improves lifelong persistent behavioral and epigenetic changes induced by early-life stress. J. Psychiatr. Res. 138, 107–116. 10.1016/j.jpsychires.2021.04.008 33848966 PMC10494235

[B19] Boschen K. E. Hamilton G. F. Delorme J. E. Klintsova A. Y. (2014). Activity and social behavior in a complex environment in rats neonatally exposed to alcohol. Alcohol 48, 533–541. 10.1016/j.alcohol.2014.07.005 25150044 PMC4163121

[B20] Boschen K. E. McKeown S. E. Roth T. L. Klintsova A. Y. (2017). Impact of exercise and a complex environment on hippocampal dendritic morphology, Bdnf gene expression and DNA methylation in male rat pups neonatally exposed to alcohol. Dev. Neurobiol. 77, 708–725. 10.1002/dneu.22448 27597545 PMC5997455

[B21] Boschen K. E. Ruggiero M. J. Klintsova A. Y. (2016). Neonatal binge alcohol exposure increases microglial activation in the developing rat hippocampus. Neuroscience 324, 355–366. 10.1016/j.neuroscience.2016.03.033 26996510 PMC4838517

[B22] Bosworth A. P. Allen N. J. (2017). The diverse actions of astrocytes during synaptic development. Curr. Opin. Neurobiol. 47, 38–43. 10.1016/j.conb.2017.08.017 28938161

[B23] Bourgeois J.-P. Goldman-Rakic P. S. Rakic P. (1994). Synaptogenesis in the prefrontal cortex of rhesus monkeys. Cereb. Cortex 4, 78–96. 10.1093/cercor/4.1.78 8180493

[B24] Brenhouse H. C. Danese A. Grassi-Oliveira R. (2018). Neuroimmune impacts of early-life stress on development and psychopathology. Curr. Top. Behav. Neurosci. 43, 423–447. 10.1007/7854_2018_53 30003509

[B25] Buchanan J. Elabbady L. Collman F. Jorstad N. L. Bakken T. E. Ott C.. (2021). Oligodendrocyte precursor cells prune axons in the mouse neocortex. bioRxiv [Preprint]. 10.1101/2021.05.29.446047 PMC9889886 36417438

[B26] Burenkova O. V. Aleksandrova E. A. Zarayskaya I. Y. (2019). Effects of early-life stress and HDAC inhibition on maternal behavior in mice. Behav. Neurosci. 133, 39–49. 10.1037/bne0000284 30489135

[B27] Bushong E. A. Martone M. E. Ellisman M. H. (2004). Maturation of astrocyte morphology and the establishment of astrocyte domains during postnatal hippocampal development. Int. J. Dev. Neurosci. 22, 73–86. 10.1016/j.ijdevneu.2003.12.008 15036382

[B28] Caballero A. Tseng K. Y. (2016). GABAergic function as a limiting factor for prefrontal maturation during adolescence. Trends Neurosci. 39, 441–448. 10.1016/j.tins.2016.04.010 27233681 PMC4930717

[B29] Calabro F. J. Murty V. P. Jalbrzikowski M. Tervo-Clemmens B. Luna B. (2020). Development of hippocampal-prefrontal cortex interactions through adolescence. Cereb. Cortex 30, 1548–1558. 10.1093/cercor/bhz186 31670797 PMC7132933

[B30] Calem M. Bromis K. McGuire P. Morgan C. Kempton M. J. (2017). Meta-analysis of associations between childhood adversity and hippocampus and amygdala volume in non-clinical and general population samples. NeuroImage Clin. 14, 471–479. 10.1016/j.nicl.2017.02.016 28275547 PMC5331153

[B31] Cameron H. A. Hazel T. G. McKay R. D. G. (1998). Regulation of neurogenesis by growth factors and neurotransmitters. J. Neurobiol. 36, 287–306. 9712310

[B32] Catale C. Gironda S. Lo Iacono L. Carola V. (2020). Microglial function in the effects of early-life stress on brain and behavioral development. J. Clin. Med. 9:468. 10.3390/jcm9020468 32046333 PMC7074320

[B33] Champagne F. A. (2013). Early environments, glucocorticoid receptors and behavioral epigenetics. Behav. Neurosci. 127, 628–636. 10.1037/a0034186 24128352

[B34] Chechik G. Meilijson I. Ruppin E. (1999). Neuronal regulation: a mechanism for synaptic pruning during brain maturation. Neural Comput. 11, 2061–2080. 10.1162/089976699300016089 10578044

[B35] Cheli V. T. Santiago González D. A. Spreuer V. Paez P. M. (2015). Voltage-gated Ca++ entry promotes oligodendrocyte progenitor cell maturation and myelination *in vitro*. Exp. Neurol. 265, 69–83. 10.1016/j.expneurol.2014.12.012 25542980 PMC4711374

[B36] Chung W.-S. Allen N. J. Eroglu C. (2015). Astrocytes Control synapse formation, function and elimination. Cold Spring Harb. Perspect. Biol. 7:a020370. 10.1101/cshperspect.a020370 25663667 PMC4527946

[B37] Coghlan S. Horder J. Inkster B. Mendez M. A. Murphy D. G. Nutt D. J. (2012). GABA system dysfunction in autism and related disorders: from synapse to symptoms. Neurosci. Biobehav. Rev. 36, 2044–2055. 10.1016/j.neubiorev.2012.07.005 22841562 PMC4477717

[B38] Conway G. D. O’Bara M. A. Vedia B. H. Pol S. U. Sim F. J. (2012). Histone deacetylase activity is required for human oligodendrocyte progenitor differentiation. Glia 60, 1944–1953. 10.1002/glia.22410 22927334

[B39] Cui Q. L. D’Abate L. Fang J. Leong S. Y. Ludwin S. Kennedy T. E.. (2012). Human fetal oligodendrocyte progenitor cells from different gestational stages exhibit substantially different potential to myelinate. Stem Cells Dev. 21, 1831–1837. 10.1089/scd.2011.0494 22122665

[B40] Dahmen B. Puetz V. B. Scharke W. Von Polier G. G. Herpertz-Dahlmann B. Konrad K. (2018). Effects of early-life adversity on hippocampal structures and associated hpa axis functions. Dev. Neurosci. 40, 13–22. 10.1159/000484238 29237154

[B41] Dandi E. Kalamari A. Touloumi O. Lagoudaki R. Nousiopoulou E. Simeonidou C.. (2018). Beneficial effects of environmental enrichment on behavior, stress reactivity and synaptophysin/BDNF expression in hippocampus following early life stress. Int. J. Dev. Neurosci. 67, 19–32. 10.1016/j.ijdevneu.2018.03.003 29545098

[B42] Davalos D. Grutzendler J. Yang G. Kim J. V. Zuo Y. Jung S.. (2005). ATP mediates rapid microglial response to local brain injury *in vivo*. Nat. Neurosci. 8, 752–758. 10.1038/nn1472 15895084

[B43] Delli Pizzi S. Padulo C. Brancucci A. Bubbico G. Edden R. A. Ferretti A.. (2016). GABA content within the ventromedial prefrontal cortex is related to trait anxiety. Soc. Cogn. Affect. Neurosci. 11, 758–766. 10.1093/scan/nsv155 26722018 PMC4847694

[B44] Delpech J. C. Wei L. Hao J. Yu X. Madore C. Butovsky O.. (2016). Early life stress perturbs the maturation of microglia in the developing hippocampus. Brain. Behav. Immun. 57, 79–93. 10.1016/j.bbi.2016.06.006 27301858 PMC5010940

[B45] DePasquale C. E. Raby K. L. Hoye J. Dozier M. (2018). Parenting predicts Strange Situation cortisol reactivity among children adopted internationally. Psychoneuroendocrinology 89, 86–91. 10.1016/j.psyneuen.2018.01.003 29334626 PMC5878708

[B301] Diorio D. Viau V. Meaney M. J. (1993). The role of the medial prefrontal cortex (cingulate gyrus) in the regulation of hypothalamic-pituitary-adrenal responses to stress. J. Neurosci. 13, 3839–3847. 10.1523/JNEUROSCI.13-09-03839.1993 8396170 PMC6576467

[B46] do Prado C. H. Narahari T. Holland F. H. Lee H. N. Murthy S. K. Brenhouse H. C. (2016). Effects of early adolescent environmental enrichment on cognitive dysfunction, prefrontal cortex development and inflammatory cytokines after early life stress. Dev. Psychobiol. 58, 482–491. 10.1002/dev.21390 26688108 PMC12878819

[B47] Dobbing J. Sands J. (1973). Q40antitative growth and development of human brain. Arch. Dis. Child. 48, 757–767. 10.1136/adc.48.10.757 4796010 PMC1648530

[B48] Dobbing J. Sands J. (1979). Comparative aspects of the brain growth spurt. Early Hum. Dev. 3, 79–83. 10.1016/0378-3782(79)90022-7 118862

[B49] Doherty T. (2019). Characterization of adversity-induced phenotypic outcomes and prevention of adversity-induced bdnf methylation *via* pharmacological manipulation of the epigenome. Doctoral dissertation-ProQuest. Available online at: https://udspace.udel.edu/bitstream/handle/19716/24740/Doherty_udel_0060D_13689.pdf?isAllowed=y&sequence=1.

[B50] Downes N. Mullins P. (2014). The development of myelin in the brain of the juvenile rat. Toxicol. Pathol. 42, 913–922. 10.1177/0192623313503518 24129760

[B51] Dozier M. Peloso E. Lindhiem O. Gordon M. K. Manni M. Sepulveda S.. (2006). Developing evidence-based interventions for foster children: an example of a randomized clinical trial with infants and toddlers. J. Soc. Issues 62, 767–785. 10.1111/j.1540-4560.2006.00486.x

[B52] Drzewiecki C. M. Juraska J. M. (2020). The structural reorganization of the prefrontal cortex during adolescence as a framework for vulnerability to the environment. Pharmacol. Biochem. Behav. 199:173044. 10.1016/j.pbb.2020.173044 33035531

[B53] Drzewiecki C. M. Willing J. Juraska J. M. (2016). Synaptic number changes in the medial prefrontal cortex across adolescence in male and female rats: a role for pubertal onset. Synapse 70, 361–368. 10.1002/syn.21909 27103097 PMC4945496

[B54] Etkin A. Wager T. D. (2007). Functional neuroimaging of anxiety: Smeta-ana lysis of emotional processing in PTSD, social anxiety disorder and specific phobia. Am. J. Psychiatry 164, 1476–1488. 10.1176/appi.ajp.2007.07030504 17898336 PMC3318959

[B55] Fair D. A. Cohen A. L. Dosenbach N. U. F. Church J. A. Miezin F. M. Barch D. M.. (2008). The maturing architecture of the brain’s default network. Proc. Natl. Acad. Sci. U. S. A. 105, 4028–4032. 10.1073/pnas.0800376105 18322013 PMC2268790

[B56] Faraco G. Pittelli M. Cavone L. Fossati S. Porcu M. Mascagni P.. (2009). Histone deacetylase (HDAC) inhibitors reduce the glial inflammatory response *in vitro* and *in vivo*. Neurobiol. Dis. 36, 269–279. 10.1016/j.nbd.2009.07.019 19635561

[B57] Farhy-Tselnicker I. Allen N. J. (2018). Astrocytes, neurons, synapses: a tripartite view on cortical circuit development. Neural Dev. 13:7. 10.1186/s13064-018-0104-y 29712572 PMC5928581

[B58] Faust T. E. Gunner G. Schafer D. P. (2021). Mechanisms governing activity-dependent synaptic pruning in the developing mammalian CNS. Nat. Rev. Neurosci. 22, 657–673. 10.1038/s41583-021-00507-y 34545240 PMC8541743

[B59] Fernandez F. Garner C. C. (2007). Over-inhibition: a model for developmental intellectual disability. Trends Neurosci. 30, 497–503. 10.1016/j.tins.2007.07.005 17825437

[B60] Field E. J. (1955). Observations on the development of microglia together with a note on the influence of cortisone. J. Anat. 89, 201–208. 14367215 PMC1244782

[B61] Fisher P. A. Ellis B. H. Chamberlain P. (1999). Early intervention foster care: a model for preventing risk in young children who have been maltreated. Child. Serv. 2, 159–182. 10.1207/s15326918cs0203_3

[B62] Fisher P. A. Gunnar M. R. Dozier M. Bruce J. Pears K. C. (2006). Effects of therapeutic interventions for foster children on behavioral problems, caregiver attachment and stress regulatory neural systems. Ann. N Y Acad. Sci. 1094, 215–225. 10.1196/annals.1376.023 17347353

[B63] Fisher P. A. Van Ryzin M. J. Gunnar M. R. (2011). Mitigating HPA axis dysregulation associated with placement changes in foster care. Psychoneuroendocrinology 36, 531–539. 10.1016/j.psyneuen.2010.08.007 20888698 PMC3610565

[B64] Fletcher J. L. Murray S. S. Xiao J. (2018). Brain-derived neurotrophic factor in central nervous system myelination: a new mechanism to promote myelin plasticity and repair. Int. J. Mol. Sci. 19:4131. 10.3390/ijms19124131 30572673 PMC6321406

[B65] Forbes T. A. Gallo V. (2017). All wrapped up: environmental effects on myelination. Trends Neurosci. 40, 572–587. 10.1016/j.tins.2017.06.009 28844283 PMC5671205

[B66] French J. A. Carp S. B. (2016). Early-life social adversity and developmental processes in nonhuman primates. Curr. Opin. Behav. Sci. 7, 40–46. 10.1016/j.cobeha.2015.11.004 26858971 PMC4742359

[B67] Fuxe K. Cintra A. Agnati L. F. Härfstrand A. Wikstrom A. C. Okret S.. (1987). Studies on the cellular localization and distribution of glucocorticoid receptor and estrogen receptor immunoreactivity in the central nervous system of the rat and their relationship to the monoaminergic and peptidergic neurons of the brain. J. Steroid Biochem. 27, 159–170. 10.1016/0022-4731(87)90306-2 2891875

[B68] Gee D. G. Gabard-Durnam L. J. Flannery J. Goff B. Humphreys K. L. Telzer E. H.. (2013). Early developmental emergence of human amygdala-prefrontal connectivity after maternal deprivation. Proc. Natl. Acad. Sci. U S A 110, 15638–15643. 10.1073/pnas.1307893110 24019460 PMC3785723

[B69] Genzel L. Dresler M. Cornu M. Jäger E. Konrad B. Adamczyk M.. (2015). Medial prefrontal-hippocampal connectivity and motor memory consolidation in depression and schizophrenia. Biol. Psychiatry 77, 177–186. 10.1016/j.biopsych.2014.06.004 25037555

[B70] Geuze E. Vermetten E. Bremner J. D. (2005). MR-based *in vivo* hippocampal volumetrics: 2. Findings in neuropsychiatric disorders. Mol. Psychiatry 10, 160–184. 10.1038/sj.mp.4001579 15356639

[B303] Ghosal S. Hare B. D. Duman R. S. (2017). Prefrontal cortex GABAergic deficits and circuit dysfunction in the pathophysiology and treatment of chronic stress and depression. Curr. Opin. Behav. Sci. 14, 1–8. 10.1016/j.cobeha.2016.09.012 27812532 PMC5086803

[B71] Giera S. Luo R. Ying Y. Ackerman S. D. Jeong S. J. Stoveken H. M.. (2018). Microglial transglutaminase-2 drives myelination and myelin repair *via* GPR56/ADGRG1 in oligodendrocyte precursor cells. eLife 7:e33385. 10.7554/eLife.33385 29809138 PMC5980231

[B72] Ginhoux F. Prinz M. (2015). Origin of microglia: current concepts and past controversies. Cold Spring Harb. Perspect. Biol. 7:a020537. 10.1101/cshperspect.a020537 26134003 PMC4526747

[B73] Giulian D. Young D. G. Woodward J. Brown D. C. Lachman L. B. (1988a). Interleukin-1 is an astroglial growth factor in the developing brain. J. Neurosci. 8, 709–714. 10.1523/JNEUROSCI.08-02-00709.1988 3257519 PMC6569312

[B74] Giulian D. Woodward J. Young G. Krebs F. Lachman B. (1988b). Interleukin-1 injected and neovascularization into mammalian brain stimulates astrogliosis and neovascularization. J. Neurosci. 8, 2485–2490. 10.1523/JNEUROSCI.08-07-02485.1988 2470873 PMC6569517

[B75] Gonzalez-Pardo H. Arias J. Gomez-Lazaro E. Taboada I. Conejo N. (2020). Sex-specific effects of early life stress on brain and neuroinflammation. Brain Sci. 10, 8–11. 10.3390/brainsci10070447. PMC7408325 32674298

[B304] Green J. G. McLaughlin K. A. Berglund P. A. Gruber M. J. Sampson N. A. Zaslavsky A. M.. (2010). Childhood adversities and adult psychiatric disorders in the national comorbidity survey replication I. Associations with first onset of DSM-IV disorders. Arch. Gen. Psychiatry 67, 113–123. 10.1001/archgenpsychiatry.2009.186 20124111 PMC2822662

[B76] Gorka A. X. Hanson J. L. Radtke S. R. Hariri A. R. (2014). Reduced hippocampal and medial prefrontal gray matter mediate the association between reported childhood maltreatment and trait anxiety in adulthood and predict sensitivity to future life stress. Biol. Mood Anxiety Disord. 4:12. 10.1186/2045-5380-4-12 25408863 PMC4236295

[B77] Guan J. Ding Y. Rong Y. Geng Y. Lai L. Q28 D.. (2020). Early life stress increases brain glutamate and induces neurobehavioral manifestations in rats. ACS Chem. Neurosci. 11, 4169–4178. 10.1021/acschemneuro.0c00454 33179901

[B78] Gunnar M. R. DePasquale C. E. Reid B. M. Donzella B. (2019). Pubertal stress recalibration reverses the effects of early life stress in postinstitutionalized children. Proc. Natl. Acad. Sci. U S A 116, 23984–23988. 10.1073/pnas.1909699116 31712449 PMC6883847

[B79] Gunnar M. R. Hostinar C. E. Sanchez M. M. Tottenham N. Sullivan R. M. (2015). Parental buffering of fear and stress neurobiology: reviewing parallels across rodent, monkey and human models. Soc. Neurosci. 10, 474–478. 10.1080/17470919.2015.1070198 26234160 PMC5198892

[B80] Gursky Z. H. Johansson J. R. Klintsova A. Y. (2020). Postnatal alcohol exposure and adolescent exercise have opposite effects on cerebellar microglia in rat. Int. J. Dev. Neurosci. 80, 558–571. 10.1002/jdn.10051 32681672 PMC8246316

[B81] Hambrick E. P. Brawner T. W. Perry B. D. (2019). Timing of early-life stress and the development of brain-related capacities. Front. Behav. Neurosci. 13:183. 10.3389/fnbeh.2019.00183 31447660 PMC6691036

[B82] Hamilton G. F. Boschen K. E. Goodlett C. R. Greenough W. T. Klintsova A. Y. (2012). Housing in environmental complexity following wheel running augments survival of newly generated hippocampal neurons in a rat model of binge alcohol exposure during the third trimester equivalent. Alcohol. Clin. Exp. Res. 36, 1196–1204. 10.1111/j.1530-0277.2011.01726.x 22324755 PMC4543282

[B83] Hamilton G. F. Jablonski S. A. Schiffino F. L. St. Cyr S. A. Stanton M. E. Klintsova A. Y. (2014). Exercise and environment as an intervention for neonatal alcohol effects on hippocampal adult neurogenesis and learning. Neuroscience 265, 274–290. 10.1016/j.neuroscience.2014.01.061 24513389 PMC4005875

[B84] Han Y. Zhang L. Wang Q. Zhang D. Zhao Q. Zhang J.. (2019). Minocycline inhibits microglial activation and alleviates depressive-like behaviors in male adolescent mice subjected to maternal separation. Psychoneuroendocrinology 107, 37–45. 10.1016/j.psyneuen.2019.04.021 31078757

[B85] Hanson J. L. Knodt A. R. Brigidi B. D. Hariri A. R. (2015). Lower structural integrity of the uncinate fasciculus is associated with a history of child maltreatment and future psychological vulnerability to stress. Dev. Psychopathol. 27, 1611–1619. 10.1017/S0954579415000978 26535947 PMC4698331

[B305] Hensch T. K. (2004). Critical period regulation. Annu. Rev. Neurosci. 27, 549–579. 10.1146/annurev.neuro.27.070203.144327 15217343

[B306] Herman J. P. (2020). Corticolimbic stress regulatory circuits, hypothalamo–pituitary–adrenocortical adaptation and resilience. Stress Resil. Mol. Behav. Aspects 291–309. 10.1016/B978-0-12-813983-7.00019-7

[B86] Hinwood M. Morandini J. Day T. A. Walker F. R. (2012). Evidence that microglia mediate the neurobiological effects of chronic psychological stress on the medial prefrontal cortex. Cereb. Cortex 22, 1442–1454. 10.1093/cercor/bhr229 21878486

[B87] Hirabayashi Y. Gotoh Y. (2010). Epigenetic control of neural precursor cell fate during development. Nat. Rev. Neurosci. 11, 377–388. 10.1038/nrn2810 20485363

[B88] Holmes A. Wellman C. L. (2009). Stress-induced prefrontal reorganization and executive dysfunction in rodents. Neurosci. Biobehav. Rev. 33, 773–783. 10.1016/J.NEUBIOREV.2008.11.005 19111570 PMC2941982

[B89] Honeycutt J. A. Demaestri C. Peterzell S. Silveri M. M. Cai X. Kulkarni P.. (2020). Altered corticolimbic connectivity reveals sex-specific adolescent outcomes in a rat model of early life adversity. eLife 9:e52651. 10.7554/eLife.52651 31958061 PMC7010412

[B90] Hostinar C. E. Stellern S. A. Schaefer C. Carlson S. M. Gunnar M. R. (2012). Associations between early life adversity and executive function in children adopted internationally from orphanages. Proc. Natl. Acad. Sci. U S A 109, 17208–17212. 10.1073/pnas.1121246109 23047689 PMC3477377

[B91] Hoye J. R. Cheishvili D. Yarger H. A. Roth T. L. Szyf M. Dozier M. (2020). Preliminary indications that the Attachment and Biobehavioral Catch-up Intervention alters DNA methylation in maltreated children. Dev. Psychopathol. 32, 1486–1494. 10.1017/S0954579419001421 31854285

[B92] Hubel D. H. Wiesel T. (1962). Receptive fields, binocuar interaction and functional architecture in the cat’s visual cortexcart. J. Physiol. 160, 106–154. 14449617 10.1113/jphysiol.1962.sp006837 PMC1359523

[B93] Hughes A. N. Appel B. (2020). Microglia phagocytose myelin sheaths to modify developmental myelination. Nat. Neurosci. 23, 1055–1066. 10.1038/s41593-020-0654-2 32632287 PMC7483351

[B94] Huttenlocher P. R. Dabholkar A. S. (1997). Regional differences in synaptogenesis in human cerebral cortex. J. Comp. Neurol. 387, 167–178. 10.1002/(sici)1096-9861(19971020)387:2<167::aid-cne1>3.0.co;2-z 9336221

[B95] Islam M. R. Luo R. Valaris S. Haley E. B. Takase H. Chen Y. I.. (2020). Diffusion tensor-MRI detects exercise-induced neuroplasticity in the hippocampal microstructure in mice. Brain Plast. 5, 147–159. 10.3233/BPL-190090 33282678 PMC7685674

[B96] Jankord R. Herman J. P. (2009). Limbic regulation of hypothalamo-pituitary-adrenocortical function during acute and chronic stress. Ann. N Y Acad. Sci. 1148, 64–73. 10.1196/annals.1410.012 19120092 PMC2637449

[B97] Jauregui-Huerta F. Ruvalcaba-Delgadillo Y. Gonzalez-Perez O. Gonzalez-Castaneda R. Garcia-Estrada J. Luquin S. (2010). Responses of glial cells to stress and glucocorticoids. Curr. Immunol. Rev. 6, 195–204. 10.2174/157339510791823790 20729991 PMC2924577

[B98] Jin J. Maren S. (2015). Prefrontal-hippocampal interactions in memory and emotion. Front. Syst. Neurosci. 9:170. 10.3389/fnsys.2015.00170 26696844 PMC4678200

[B99] Johe K. K. Hazel T. G. Muller T. Dugich-Djordjevic M. M. McKay R. D. G. (1996). Single factors direct the differentiation of stem cells from the fetal and adult central nervous system. Genes Dev. 10, 3129–3140. 10.1101/gad.10.24.3129 8985182

[B100] Johnson F. K. Kaffman A. (2018). Early life stress perturbs the function of microglia in the developing rodent brain: new insights and future challenges. Brain. Behav. Immun. 69, 18–27. 10.1016/j.bbi.2017.06.008 28625767 PMC5732099

[B101] Kambeitz C. Klug M. G. Greenmyer J. Popova S. Burd L. (2019). Association of adverse childhood experiences and neurodevelopmental disorders in people with fetal alcohol spectrum disorders (FASD) and non-FASD controls. BMC Pediatr. 19:498. 10.1186/s12887-019-1878-8 31842817 PMC6912946

[B102] Keller S. M. Nowak A. Roth T. L. (2019). Female pups receive more maltreatment from stressed dams. Dev. Psychobiol. 61, 824–831. 10.1002/dev.21834 30810229 PMC6711830

[B103] Kempermann G. Gage F. H. (1999). Experience-dependent regulation of adult hippocampal neurogenesis: effects of long-term stimulation and stimulus withdrawal. Hippocampus 9, 321–332. 10.1002/(SICI)1098-1063(1999)9:3<321::AID-HIPO11>3.0.CO;2-C 10401646

[B104] Kentner A. C. (2015). Neuroprotection and recovery from early-life adversity: considerations for environmental enrichment. Neural Regen. Res. 10, 1545–1547. 10.4103/1673-5374.165315 26692834 PMC4660730

[B105] Khalaf-Nazzal R. Francis F. (2013). Hippocampal development - Old and new findings. Neuroscience 248, 225–242. 10.1016/j.neuroscience.2013.05.061 23756184

[B106] Kim J. Yoon K. S. (1998). Stress: metaplastic effects in the hippocampus. Trends Neurosci. 21, 505–509. 10.1016/s0166-2236(98)01322-8 9881846

[B107] Kitayama N. Vaccarino V. Kutner M. Weiss P. Bremner J. D. (2005). Magnetic resonance imaging (MRI) measurement of hippocampal volume in posttraumatic stress disorder: A meta-analysis. J. Affect. Disord. 88, 79–86. 10.1016/j.jad.2005.05.014 16033700

[B108] Knudsen E. I. (2004). Sensitive periods in the development of the brain and behavior. J. Cogn. Neurosci. 16, 1412–1425. 10.1162/0898929042304796 15509387

[B109] Kohman R. A. Bhattacharya T. K. Wojcik E. Rhodes J. S. (2013). Exercise reduces activation of microglia isolated from hippocampus and brain of aged mice. J. Neuroinflammation 10:114. 10.1186/1742-2094-10-114 24044641 PMC3848770

[B110] Koss W. A. Belden C. E. Hristov A. D. Juraska J. M. (2014). Dendritic remodeling in the adolescent medial prefrontal cortex and the basolateral amygdala of male and female rats. Synapse 68, 61–72. 10.1002/syn.21716 24105875

[B111] Kukley M. Nishiyama A. Dietrich D. (2010). The fate of synaptic input to NG2 glial cells: Neurons specifically downregulate transmitter release onto differentiating oligodendroglial cells. J. Neurosci. 30, 8320–8331. 10.1523/JNEUROSCI.0854-10.2010 20554883 PMC6634580

[B112] Kumar S. Cole R. Chiappelli F. Vellis J. D. (1989). Differential regulation of oligodendrocyte markers by glucocorticoids: post-transcriptional regulation of both proteolipid protein and myelin basic protein and transcriptional regulation of glycerol phosphate dehydrogenase. Proc. Natl. Acad. Sci. U S A 86, 6807–6811. 10.1073/pnas.86.17.6807 2475873 PMC297935

[B113] Kwak H. R. Lei J. W. Kwon K. J. Kang C. D. Cheong Y. Chun W.. (2009). Maternal social separation of adolescent rats induces hyperactivity and anxiolytic behavior. Korean J. Physiol. Pharmacol. 13, 79–83. 10.4196/kjpp.2009.13.2.79 19885001 PMC2766697

[B114] Kwon D. Pfefferbaum A. Sullivan E. V. Pohl K. M. (2020). Regional growth trajectories of cortical myelination in adolescents and young adults: longitudinal validation and functional correlates. Brain Imaging Behav. 14, 242–266. 10.1007/s11682-018-9980-3 30406353 PMC6506406

[B307] Lajud N. Roque A. Cajero M. Gutiérrez-Ospina G. Torner L. (2012). Periodic maternal separation decreases hippocampal neurogenesis without affecting basal corticosterone during the stress hyporesponsive period, but alters HPA axis and coping behavior in adulthood. Psychoneuroendocrinology 37, 410–420. 10.1016/j.psyneuen.2011.07.011 21862224

[B115] Lambert H. K. Peverill M. Sambrook K. A. Rosen M. L. Sheridan M. A. McLaughlin K. A. (2019). Altered development of hippocampus-dependent associative learning following early-life adversity. Dev. Cogn. Neurosci. 38:100666. 10.1016/j.dcn.2019.100666 31276941 PMC6684815

[B116] Lawson L. J. Perry V. H. Dri P. Gordon S. (1990). Heterogeneity in the distribution and morphology of microglia in the normal adult mouse brain. Neuroscience 39, 151–170. 10.1016/0306-4522(90)90229-w 2089275

[B117] Leardini-Tristaõ M. Andrade G. Garcia C. Reis P. A. Lourenço M. Moreira E. T. S.. (2020). Physical exercise promotes astrocyte coverage of microvessels in a model of chronic cerebral hypoperfusion. J. Neuroinflammation 17:117. 10.1186/s12974-020-01771-y 32299450 PMC7161182

[B119] Lebel C. Beaulieu C. (2011). Longitudinal development of human brain wiring continues from childhood into adulthood. J. Neurosci. 31, 10937–10947. 10.1523/JNEUROSCI.5302-10.2011 21795544 PMC6623097

[B118] Lebel C. A. McMorris C. A. Kar P. Ritter C. Andre Q. Tortorelli C.. (2019). Characterizing adverse prenatal and postnatal experiences in children. Birth Defects Res. 111, 848–858. 10.1002/bdr2.1464 30690931

[B120] Lee J. C. Mayer-Proschel M. Rao M. S. (2000). Gliogenesis in the central nervous system. Glia 30, 105–121. 10.1002/(sici)1098-1136(200004)30:2<105::aid-glia1>3.0.co;2-h 10719353

[B121] Levison S. W. Chuang C. Abramson B. J. Goldman J. E. (1993). The migrational patterns and developmental fates of glial precursors in the rat subventricular zone are temporally regulated. Development 119, 611–622. 8187632 10.1242/dev.119.3.611

[B122] Li Y. Luo Y. Tang J. Liang X. Wang J. Xiao Q.. (2021). The positive effects of running exercise on hippocampal astrocytes in a rat model of depression. Transl. Psychiatry 11:83. 10.1038/s41398-021-01216-x 33526783 PMC7851162

[B123] Ling E. A. (1982). Influence of cortisone on amoeboid microglia and microglial cells in the corpus callosum in postnatal rats. J. Anat. 134, 705–771. 7130035 PMC1167865

[B124] Liston C. Gan W. B. (2011). Glucocorticoids are critical regulators of dendritic spine development and plasticity *in vivo*. Proc. Natl. Acad. Sci. U S A 108, 16074–16079. 10.1073/pnas.1110444108 21911374 PMC3179117

[B125] Llorente R. Gallardo M. L. Berzal A. L. Prada C. Garcia-Segura L. M. Viveros M. P. (2009). Early maternal deprivation in rats induces gender-dependent effects on developing hippocampal and cerebellar cells. Int. J. Dev. Neurosci. 27, 233–241. 10.1016/j.ijdevneu.2009.01.002 19429388

[B308] Loman M. M. Gunnar M. R. (2010). Early experience and the development of stress reactivity and regulation in children. Neurosci. Biobehav. Rev. 34, 867–876. 10.1016/j.neubiorev.2009.05.007 19481109 PMC2848877

[B126] López-Murcia F. J. Terni B. Llobet A. (2015). SPARC triggers a cell-autonomous program of synapse elimination. Proc. Natl. Acad. Sci. U S A 112:201512202. 10.1073/pnas.1512202112 PMC4629317 26420865

[B127] Lovic V. Fleming A. S. (2004). Artificially-reared female rats show reduced prepulse inhibition and deficits in the attentional set shifting task—reversal of effects with maternal-like licking stimulation. Behav. Brain Res. 148, 209–219. 10.1016/s0166-4328(03)00206-7 14684261

[B128] Lu B. Nagappan G. Lu Y. (2014). “BDNF and synaptic plasticity, cognitive function and dysfunction,” in Neurotrophic Factors. Handbook of Experimental Pharmacology, eds Lewin G. R. Carter B. D. (Berlin, Heidelberg: Springer Berlin Heidelberg), 223–250. 10.1007/978-3-642-45106-5_9 24668475

[B129] Lu Q. R. Yuk D. I. Alberta J. A. Zhu Z. Pawlitzky I. Chan J.. (2000). Sonic hedgehog-regulated oligodendrocyte lineage genes encoding bhlh proteins in the mammalian central nervous system. Neuron 25, 317–329. 10.1016/s0896-6273(00)80897-1 10719888

[B130] Makinodan M. Rosen K. M. Ito S. Corfas G. (2012). A critical period for social experience-dependent oligodendrocyte maturation and myelination. Science 337, 1357–1360. 10.1126/science.1220845 22984073 PMC4165613

[B131] Mallya A. P. Wang H.-D. Lee H. N. R. Deutch A. Y. (2019). Microglial pruning of synapses in the prefrontal cortex during adolescence. Cereb. Cortex 29, 1634–1643. 10.1093/cercor/bhy061 29668872 PMC6418387

[B132] Maniam J. Antoniadis C. Morris M. J. (2014). Early-life stress, HPA axis adaptation and mechanisms contributing to later health outcomes. Front. Endocrinol. (Lausanne). 5:73. 10.3389/fendo.2014.00073 24860550 PMC4026717

[B133] Mauch D. H. Nägier K. Schumacher S. Göritz C. Müller E. C. Otto A.. (2001). CNS synaptogenesis promoted by glia-derived cholesterol. Science 294, 1354–1357. 10.1126/science.294.5545.1354 11701931

[B134] McKinnon R. D. Waldron S. Kiel M. E. (2005). PDGF α-receptor signal strength controls an RTK rheostat that integrates phosphoinositol 3′-kinase and phospholipase Cγ pathways during oligodendrocyte maturation. J. Neurosci. 25, 3499–3508. 10.1523/JNEUROSCI.5049-04.2005 15814780 PMC6725367

[B135] Meaney M. J. Diorio J. Francis D. Widdowson J. LaPlante P. Caldji C.. (1996). Early environmental regulation of forebrain glucocorticoid receptor gene expression: implications for adrenocortical responses to stress. Dev. Neurosci. 18, 61–72. 10.1159/000111395 8840086

[B136] Mee-inta O. Zhao Z.-W. Kuo Y.-M. (2019). Physical exercise inhibits inflammation and microglial activation. Cells 8:691. 10.3390/cells8070691 31324021 PMC6678635

[B137] Melliar-Smith P. M. Moser L. E. (2015). “Conversion infrastructure for maintaining high availability of web services using multiple service providers,” in IEEE 2015 IEEE International Conference on Web Services (ICWS), 2017, (New York, NY), 759–764. 10.1109/ICWS.2015.110

[B138] Meyer N. Rinholm J. E. (2021). Mitochondria in myelinating oligodendrocytes: Slow and out of breath? Metabolites 11:359. 10.3390/metabo11060359 34198810 PMC8226700

[B139] Miller D. J. Duka T. Stimpson C. D. Schapiro S. J. Baze W. B. McArthur M. J.. (2012). Prolonged myelination in human neocortical evolution. Proc. Natl. Acad. Sci. U S A 109, 16480–16485. 10.1073/pnas.1117943109 23012402 PMC3478650

[B140] Miller E. K. Cohen J. D. (2001). An integrative theory of prefrontal cortex function. Annu. Rev. Neurosci. 24, 167–202. 10.1146/annurev.neuro.24.1.167 11283309

[B141] Monninger M. Kraaijenvanger E. J. Pollok T. M. Boecker-Schlier R. Jennen-Steinmetz C. Baumeister S.. (2020). The long-term impact of early life stress on orbitofrontal cortical thickness. Cereb. Cortex 30, 1307–1317. 10.1093/cercor/bhz167 31504259

[B142] Monteiro B. M. M. Moreira F. A. Massensini A. R. Moraes M. F. D. Pereira G. S. (2014). Enriched environment increases neurogenesis and improves social memory persistence in socially isolated adult mice. Hippocampus 24, 239–248. 10.1002/hipo.22218 24123782

[B143] Moriceau S. Raineki C. Holman J. D. Holman J. G. Sullivan R. M. (2009). Enduring neurobehavioral effects of early life trauma mediated through learning and corticosterone suppression. Front. Behav. Neurosci. 3:22. 10.3389/neuro.08.022.2009 19750195 PMC2741290

[B144] Moriguchi Y. Hiraki K. (2013). Prefrontal cortex and executive function in young children: a review of NIRS studies. Front. Hum. Neurosci. 7:867. 10.3389/fnhum.2013.00867 24381551 PMC3865781

[B145] Murao N. Noguchi H. Nakashima K. (2016). Epigenetic regulation of neural stem cell property from embryo to adult. Neuroepigenetics 5, 1–10. 10.1016/j.nepig.2016.01.001

[B146] Nägler K. Mauch D. H. Pfrieger F. W. (2001). Glia-derived signals induce synapse formation in neurones of the rat central nervous system. J. Physiol. 533, 665–679. 10.1111/j.1469-7793.2001.00665.x 11410625 PMC2278670

[B147] Nakafuku M. del Águila Á. (2020). Developmental dynamics of neurogenesis and gliogenesis in the postnatal mammalian brain in health and disease: historical and future perspectives. Wiley Interdiscip. Rev. Dev. Biol. 9:e369. 10.1002/wdev.369 31825170 PMC8106805

[B148] Nakamura D. S. Lin Y. H. Khan D. Gothié J. D. M. de Faria O. Dixon J. A.. (2021). Mitochondrial dynamics and bioenergetics regulated by netrin-1 in oligodendrocytes. Glia 69, 392–412. 10.1002/glia.23905 32910475

[B149] Nelson C. A. Gabard-Durnam L. J. (2020). Early adversity and critical periods: neurodevelopmental consequences of violating the expectable environment. Trends Neurosci. 43, 133–143. 10.1016/j.tins.2020.01.002 32101708 PMC8092448

[B150] Nemes-Baran A. D. White D. R. DeSilva T. M. (2020). Fractalkine-dependent microglial pruning of viable oligodendrocyte progenitor cells regulates myelination. Cell Rep. 32:108047. 10.1016/j.celrep.2020.108047 32814050 PMC7478853

[B151] Nery S. Wichterle H. Fishell G. (2001). Sonic hedgehog contributes to oligodendrocyte specification in the mammalian forebrain. Development 128, 527–540. 10.1242/dev.128.4.527 11171336

[B152] Nguyen H.-B. Bagot R. C. Diorio J. Wong T. P. Meaney M. J. (2015). Maternal care differentially affects neuronal excitability and synaptic plasticity in the dorsal and ventral hippocampus. Neuropsychopharmacology 40, 1590–1599. 10.1038/npp.2015.19 25598429 PMC4915255

[B153] Liu P. Z. Nusslock R. (2018). How stress gets under the skin: early life adversity and glucocorticoid receptor epigenetic regulation. Curr. Genomics 19, 653–664. 10.2174/1389202919666171228164350 30532645 PMC6225447

[B154] Ohline S. M. Abraham W. C. (2018). Environmental enrichment effects on synaptic and cellular physiology of hippocampal neurons. Neuropharmacology 145, 3–12. 10.1016/j.neuropharm.2018.04.007 29634984

[B155] Pang Y. Fan L.-W. Tien L.-T. Dai X. Zheng B. Cai Z.. (2013). Differential roles of astrocyte and microglia in supporting oligodendrocyte development and myelination *in vitro*. Brain Behav. 3, 503–514. 10.1002/brb3.152 24392271 PMC3869978

[B156] Paolicelli R. C. Gross C. T. (2011). Microglia in development: linking brain wiring to brain environment. Neuron Glia Biol. 7, 77–83. 10.1017/S1740925X12000105 22857738

[B157] Paolicelli R. C. Bolasco G. Pagani F. Maggi L. Scianni M. Panzanelli P.. (2011). Synaptic pruning by microglia is necessary for normal brain development. Science 333, 1456–1458. 10.1126/science.1202529 21778362

[B309] Parellada E. Gassó P. (2021). Glutamate and microglia activation as a driver of dendritic apoptosis: a core pathophysiological mechanism to understand schizophrenia. Transl. Psychiatry 11:271. 10.1038/s41398-021-01385-9 33958577 PMC8102516

[B158] Parkhurst C. N. Yang G. Ninan I. Savas J. N. Yates J. R. Lafaille J. J.. (2013). Microglia promote learning-dependent synapse formation through brain-derived neurotrophic factor. Cell 155, 1596–1609. 10.1016/j.cell.2013.11.030 24360280 PMC4033691

[B159] Pattwell S. S. Liston C. Jing D. Ninan I. Yang R. R. Witztum J.. (2016). Dynamic changes in neural circuitry during adolescence are associated with persistent attenuation of fear memories. Nat. Commun. 7:11475. 10.1038/ncomms11475 27215672 PMC4890178

[B160] Pelkey K. A. Chittajallu R. Craig M. T. Tricoire L. Wester J. C. McBain C. J. (2017). Hippocampal gabaergic inhibitory interneurons. Physiol. Rev. 97, 1619–1747. 10.1152/physrev.00007.2017 28954853 PMC6151493

[B161] Pfeiffer S. E. Warrington A. E. Bansal R. (1993). The oligodendrocyte and its many cellular processes. Trends Cell Biol. 3, 191–197. 10.1016/0962-8924(93)90213-k 14731493

[B162] Pfrieger F. W. Barres B. A. (1996). New views on synapse-glia interactions. Curr. Opin. Neurobiol. 6, 615–621. 10.1016/s0959-4388(96)80093-6 8937825

[B163] Poletti S. Vai B. Smeraldi E. Cavallaro R. Colombo C. Benedetti F. (2016). Adverse childhood experiences influence the detrimental effect of bipolar disorder and schizophrenia on cortico-limbic grey matter volumes. J. Affect. Disord. 189, 290–297. 10.1016/j.jad.2015.09.049 26454335

[B164] Popoli M. Yan Z. McEwen B. Sanacora G. (2011). The stressed synapse: the impact of stress and glucocorticoids on glutamate transmission. Nat. Rev. Neurosci. 13, 22–37. 10.1038/nrn3138 22127301 PMC3645314

[B165] Pujol J. Vendrell P. Junqué C. Martí-Vilalta J. L. Capdevila A. (1993). When does human brain development end? Evidence of corpus callosum growth up to adulthood. Ann. Neurol. 34, 71–75. 10.1002/ana.410340113 8517683

[B166] Pusic A. D. Kraig R. P. (2014). Youth and environmental enrichment generate serum exosomes containing miR-219 that promote CNS myelination. Glia 62, 284–299. 10.1002/glia.22606 24339157 PMC4096126

[B167] Raff M. C. Lillien L. E. Richardson W. D. Burne J. F. Noble M. D. (1988). Platelet-derived growth factor from astrocytes drives the clock that times oligodendrocyte development in culture. Nature 333, 562–565. 10.1038/333562a0 3287177

[B168] Raineki C. Opendak M. Sarro E. Showler A. Bui K. McEwen B. S.. (2019). During infant maltreatment, stress targets hippocampus, but stress with mother present targets amygdala and social behavior. Proc. Natl. Acad. Sci. U S A 116, 22821–22832. 10.1073/pnas.1907170116 31636210 PMC6842629

[B169] Rajan P. McKay R. D. G. (1998). Multiple routes to astrocytic differentiation in the CNS. J. Neurosci. 18, 3620–3629. 10.1523/JNEUROSCI.18-10-03620.1998 9570793 PMC6793143

[B170] Rao U. Chen L. A. Bidesi A. S. Shad M. U. Thomas M. A. Hammen C. L. (2010). Hippocampal changes associated with early-life adversity and vulnerability to depression. Biol. Psychiatry 67, 357–364. 10.1016/j.biopsych.2009.10.017 20015483 PMC2821020

[B171] Reemst K. Noctor S. C. Lucassen P. J. Hol E. M. (2016). The indispensable roles of microglia and astrocytes during brain development. Front. Hum. Neurosci. 10:566. 10.3389/fnhum.2016.00566 27877121 PMC5099170

[B172] Reshetnikov V. Ryabushkina Y. Kovner A. Lepeshko A. Bondar N. (2020). Repeated and single maternal separation specifically alter microglial morphology in the prefrontal cortex and neurogenesis in the hippocampus of 15-day-old male mice. Neuroreport 31, 1256–1264. 10.1097/WNR.0000000000001544 33165192

[B173] Reul J. M. H. M. De Kloet E. R. (1986). Anatomical resolution of two types of corticosterone receptor sites in rat brain with *in vitro* autoradiography and computerized image analysis. J. Steroid Biochem. 24, 269–272. 10.1016/0022-4731(86)90063-4 3702410

[B174] Réus G. Z. Silva R. H. de Moura A. B. Presa J. F. Abelaira H. M. Abatti M.. (2019). Early maternal deprivation induces microglial activation, alters glial fibrillary acidic protein immunoreactivity and indoleamine 2,3-dioxygenase during the development of offspring rats. Mol. Neurobiol. 56, 1096–1108. 10.1007/s12035-018-1161-2 29873040

[B175] Richardson W. D. Pringle N. Mosley M. J. Westermark B. Dubois-Dalcg M. (1988). A role for platelet-derived growth factor in normal gliogenesis in the central nervous system. Cell 53, 309–319. 10.1016/0092-8674(88)90392-3 2834067

[B176] Robinson-Drummer P. A. Opendak M. Blomkvist A. Chan S. Tan S. Delmer C.. (2019). Infant trauma alters social buffering of threat learning: emerging role of prefrontal cortex in preadolescence. Front. Behav. Neurosci. 13:132. 10.3389/fnbeh.2019.00132 31293398 PMC6598593

[B177] Roceri M. Cirulli F. Pessina C. Peretto P. Racagni G. Riva M. A. (2004). Postnatal repeated maternal deprivation produces age-dependent changes of brain-derived neurotrophic factor expression in selected rat brain regions. Biol. Psychiatry 55, 708–714. 10.1016/j.biopsych.2003.12.011 15038999

[B178] Rosko L. Smith V. N. Yamazaki R. Huang J. K. (2019). Oligodendrocyte bioenergetics in health and disease. Neuroscientist 25, 334–343. 10.1177/1073858418793077 30122106 PMC6745601

[B179] Rossetti A. C. Papp M. Gruca P. Paladini M. S. Racagni G. Riva M. A.. (2016). Stress-induced anhedonia is associated with the activation of the inflammatory system in the rat brain: restorative effect of pharmacological intervention. Pharmacol. Res. 103, 1–12. 10.1016/j.phrs.2015.10.022 26535964

[B180] Roth T. L. Lubin F. D. Funk A. J. Sweatt J. D. (2009). Lasting epigenetic influence of early-life adversity on the BDNF gene. Biol. Psychiatry 65, 760–769. 10.1016/j.biopsych.2008.11.028 19150054 PMC3056389

[B181] Ruggiero M. J. Boschen K. E. Roth T. L. Klintsova A. Y. (2018). Sex differences in early postnatal microglial colonization of the developing rat hippocampus following a single-day alcohol exposure. J. Neuroimmune Pharmacol. 13, 189–203. 10.1007/s11481-017-9774-1 29274031 PMC5997457

[B182] Sachs B. D. Rodriguiz R. M. Siesser W. B. Kenan A. Royer E. L. Jacobsen J. P. R.. (2013). The effects of brain serotonin deficiency on behavioural disinhibition and anxiety-like behaviour following mild early life stress. Int. J. Neuropsychopharmacol. 16, 2081–2094. 10.1017/S1461145713000321 23672796 PMC3931011

[B183] Sapolsky R. M. (1996). Stress, glucocorticoids and damage to the nervous system: the current state of confusion. Stress 1, 1–19. 10.3109/10253899609001092 9807058

[B184] Sapolsky R. M. Meaney M. J. (1986). Maturation of the adrenocortical stress response: neuroendocrine control mechanisms and the stress hyporesponsive period. Brain Res. 396, 64–76. 10.1016/s0006-8993(86)80190-1 3011218

[B185] Sauvageot C. M. Stiles C. D. (2002). Molecular mechanisms controlling cortical gliogenesis. Curr. Opin. Neurobiol. 12, 244–249. 10.1016/s0959-4388(02)00322-7 12049929

[B186] Schafer D. P. Lehrman E. K. Kautzman A. G. Koyama R. Mardinly A. R. Yamasaki R.. (2012). Microglia sculpt postnatal neural circuits in an activity and complement-dependent manner. Neuron 74, 691–705. 10.1016/j.neuron.2012.03.026 22632727 PMC3528177

[B187] Schmidt M. Levine S. Oitzl M. S. van der Mark M. Müller M. B. Holsboer F.. (2005). Glucocorticoid receptor blockade disinhibits pituitary-adrenal activity during the stress hyporesponsive period of the mouse. Endocrinology 146, 1458–1464. 10.1210/en.2004-1042 15591147

[B188] Schwarz J. M. Hutchinson M. R. Bilbo S. D. (2011). Behavioral/systems/cognitive early-life experience decreases drug-induced reinstatement of morphine CPP in adulthood *via* microglial-specific epigenetic programming of anti-inflammatory IL-10 expression. J. Neurosci. 31, 17835–17847. 10.1523/JNEUROSCI.3297-11.2011 22159099 PMC3259856

[B189] Shen S. Li J. Casaccia-Bonnefil P. (2005). Histone modifications affect timing of oligodendrocyte progenitor differentiation in the developing rat brain. J. Cell Biol. 169, 577–589. 10.1083/jcb.200412101 15897262 PMC2171688

[B190] Silvers J. A. Lumian D. S. Gabard-Durnam L. Gee D. G. Goff B. Fareri D. S.. (2016). Previous institutionalization is followed by broader amygdala-hippocampal-PFC network connectivity during aversive learning in human development. J. Neurosci. 36, 6420–6430. 10.1523/JNEUROSCI.0038-16.2016 27307231 PMC5015779

[B191] Simons M. Nave K.-A. (2015). Oligodendrocytes: myelination and axonal support. Cold Spring Harb. Perspect. Biol. 8:a020479. 10.1101/cshperspect.a020479 26101081 PMC4691794

[B192] Slattery D. A. Neumann I. D. (2008). No stress please! Mechanisms of stress hyporesponsiveness of the maternal brain. J. Physiol. 586, 377–385. 10.1113/jphysiol.2007.145896 17974588 PMC2375601

[B193] Smith B. L. Morano R. L. Ulrich-Lai Y. M. Myers B. Solomon M. B. Herman J. P. (2018). Adolescent environmental enrichment prevents behavioral and physiological sequelae of adolescent chronic stress in female (but not male) rats. Stress 21, 464–473. 10.1080/10253890.2017.1402883 29166811 PMC5963965

[B194] Smith M. E. (2005). Bilateral hippocampal volume reduction in adults with post-traumatic stress disorder: a meta-analysis of structural MRI studies. Hippocampus 15, 798–807. 10.1002/hipo.20102 15988763

[B195] Sominsky L. De Luca S. Spencer S. J. (2018). Microglia: key players in neurodevelopment and neuronal plasticity. Int. J. Biochem. Cell Biol. 94, 56–60. 10.1016/j.biocel.2017.11.012 29197626

[B196] Sommer L. (2006). “Growth factors regulating neural crest cell fate decisions,” in Neural Crest Induction and Differentiation, (Vol. 589), ed Saint-Jeannet J.-P. (Boston, MA: Springer US), 197–205. 10.1007/978-0-387-46954-6_12 17076283

[B197] Sonnewald U. Westergaard N. Schousboe A. (1997). Glutamate transport and metabolism in astrocytes. Glia 21, 56–63. 10.1002/(sici)1098-1136(199709)21:1<56::aid-glia6>3.0.co;2-# 9298847

[B198] Spitzer S. O. Sitnikov S. Kamen Y. Evans K. A. Kronenberg-Versteeg D. Dietmann S.. (2019). Oligodendrocyte progenitor cells become regionally diverse and heterogeneous with age. Neuron 101, 459–471.e5. 10.1016/j.neuron.2018.12.020 30654924 PMC6372724

[B199] Stevens B. Allen N. J. Vazquez L. E. Howell G. R. Christopherson K. S. Nouri N.. (2007). The classical complement cascade mediates CNS synapse elimination. Cell 131, 1164–1178. 10.1016/j.cell.2007.10.036 18083105

[B200] Stiles J. Jernigan T. L. (2010). The basics of brain development. Neurophyschol. Rev. 20, 327–348. 10.1007/s11065-010-9148-4 21042938 PMC2989000

[B201] Sullivan R. Holman P. (2010). Transitions in sensitive period attachment learning in infancy: the role of corticosterone. Neurosci. Biobehav. Rev. 34, 835–844. 10.1016/j.neubiorev.2009.11.010 19931556 PMC2848912

[B202] Sultan S. Li L. Moss J. Petrelli F. Cassé F. Gebara E.. (2015). Synaptic integration of adult-born hippocampal neurons is locally controlled by astrocytes. Neuron 88, 957–972. 10.1016/j.neuron.2015.10.037 26606999

[B203] Tanti A. Kim J. J. Wakid M. Davoli M. A. Turecki G. Mechawar N. (2018). Child abuse associates with an imbalance of oligodendrocyte-lineage cells in ventromedial prefrontal white matter. Mol. Psychiatry 23, 2018–2028. 10.1038/mp.2017.231 29158585

[B204] Teicher M. H. Anderson C. M. Polcari A. (2012). Childhood maltreatment is associated with reduced volume in the hippocampal subfields CA3, dentate gyrus and subiculum. Proc. Natl. Acad. Sci. U S A 109, E563–E572. 10.1073/pnas.1115396109 22331913 PMC3295326

[B205] Teissier A. Le Magueresse C. Olusakin J. Andrade da Costa B. L. S. De Stasi A. M. Bacci A.. (2020). Early-life stress impairs postnatal oligodendrogenesis and adult emotional behaviour through activity-dependent mechanisms. Mol. Psychiatry 25, 1159–1174. 10.1038/s41380-019-0493-2 31439936 PMC7244403

[B206] Theoretical Empirical Practical Rationale. (2008). Monographs of the Society for Research in Child Development 73, 1–15. 10.1111/j.1540-5834.2008.00483.x

[B207] Tomlinson L. Huang P. H. Colognato H. (2018). Prefrontal cortex NG2 glia undergo a developmental switch in their responsiveness to exercise. Dev. Neurobiol. 78, 687–700. 10.1002/dneu.22590 29569358

[B208] Tomlinson L. Leiton C. V. Colognato H. (2016). Behavioral experiences as drivers of oligodendrocyte lineage dynamics and myelin plasticity. Neuropharmacology 110, 548–562. 10.1016/j.neuropharm.2015.09.016 26415537 PMC7863702

[B209] Tottenham N. (2012). Human amygdala development in the absence of species-expected caregiving. Dev. Psychobiol. 54, 598–611. 10.1002/dev.20531 22714586 PMC3404246

[B210] Traiffort E. Kassoussi A. Zahaf A. Laouarem Y. (2020). Astrocytes and microglia as major players of myelin production in normal and pathological conditions. Front. Cell. Neurosci. 14:79. 10.3389/fncel.2020.00079 32317939 PMC7155218

[B211] Traiffort E. Zakaria M. Laouarem Y. Ferent J. (2016). Hedgehog: A key signaling in the development of the oligodendrocyte lineage. J. Dev. Biol. 4:28. 10.3390/jdb4030028 29615592 PMC5831774

[B212] Treccani G. Yigit H. Lingner T. Schleuβner V. Mey F. van der Kooij M. A.. (2021). Early life adversity targets the transcriptional signature of hippocampal NG2+ glia and affects voltage gated sodium (Nav) channels properties. Neurobiol. Stress 15:100338. 10.1016/j.ynstr.2021.100338 34095364 PMC8164094

[B213] Underwood M. D. Bakalian M. J. Escobar T. Kassir S. Mann J. J. Arango V. (2019). Early-life adversity, but not suicide, is associated with less prefrontal cortex gray matter in adulthood. Int. J. Neuropsychopharmacol. 22, 349–357. 10.1093/ijnp/pyz013 30911751 PMC6499245

[B214] Van Bodegom M. Homberg J. R. Henckens M. J. A. G. (2017). Modulation of the hypothalamic-pituitary-adrenal axis by early life stress exposure. Front. Cell. Neurosci. 11:87. 10.3389/fncel.2017.00087 28469557 PMC5395581

[B215] Van Eekelen J. A. M. Jiang W. De Kloet E. R. Bohn M. C. (1988). Distribution of the mineralocorticoid and the glucocorticoid receptor mRNAs in the rat hippocampus. J. Neurosci. Res. 21, 88–94. 10.1002/jnr.490210113 2851057

[B216] van Harmelen A. L. de Jong P. J. Glashouwer K. A. Spinhoven P. Penninx B. W. J. H. Elzinga B. M. (2010). Child abuse and negative explicit and automatic self-associations: the cognitive scars of emotional maltreatment. Behav. Res. Ther. 48, 486–494. 10.1016/j.brat.2010.02.003 20303472

[B217] Van Harmelen A. L. Hauber K. Moor B. G. Spinhoven P. Boon A. E. Crone E. A.. (2014a). Childhood emotional maltreatment severity is associated with dorsal medial prefrontal cortex responsivity to social exclusion in young adults. PLoS One 9:e85107. 10.1371/journal.pone.0085107 24416347 PMC3885678

[B218] Van Harmelen A. L. Van Tol M. J. Dalgleish T. Van der Wee N. J. A. Veltman D. J. Aleman A.. (2014b). Hypoactive medial prefrontal cortex functioning in adults reporting childhood emotional maltreatment. Soc. Cogn. Affect. Neurosci. 9, 2026–2033. 10.1093/scan/nsu008 24493840 PMC4249477

[B219] van Praag H. Kempermann G. Gage F. H. (2000). Neural consequences of enviromental enrichment. Nat. Rev. Neurosci. 1, 191–198. 10.1038/35044558 11257907

[B220] Vardimon L. Ben-Dror I. Avisar N. Oren A. Shiftan L. (1999). Glucocorticoid control of glial gene expression. J. Neurobiol. 40, 513–527. 10.1002/(sici)1097-4695(19990915)40:4<513::aid-neu8>3.0.co;2-d 10453053

[B221] Vivar C. van Praag H. (2017). Running changes the brain: the long and the short of it. Physiology (Bethesda) 32, 410–424. 10.1152/physiol.00017.2017 29021361 PMC6148340

[B222] Volmar C. H. Wahlestedt C. (2015). Histone deacetylases (HDACs) and brain function. Neuroepigenetics 1, 20–27. 10.1016/j.nepig.2014.10.002

[B223] Voss M. W. Vivar C. Kramer A. F. van Praag H. (2013). Bridging animal and human models of exercise-induced brain plasticity. Trends Cogn. Sci. 17, 525–544. 10.1016/j.tics.2013.08.001 24029446 PMC4565723

[B224] Wakselman S. Béchade C. Roumier A. Bernard D. Triller A. Bessis A. (2008). Developmental neuronal death in hippocampus requires the microglial CD11b integrin and DAP12 immunoreceptor. J. Neurosci. 28, 8138–8143. 10.1523/JNEUROSCI.1006-08.2008 18685038 PMC6670768

[B225] Wang L. C. Almazan G. (2016). Role of sonic hedgehog signaling in oligodendrocyte differentiation. Neurochem. Res. 41, 3289–3299. 10.1007/s11064-016-2061-3 27639396

[B226] Wang R. Wang W. Xu J. Liu D. Wu H. Q28n X.. (2020). Jmjd3 is involved in the susceptibility to depression induced by maternal separation *via* enhancing the neuroinflammation in the prefrontal cortex and hippocampus of male rats. Exp. Neurol. 328:113254. 10.1016/j.expneurol.2020.113254 32084453

[B227] Weaver I. C. G. Cervoni N. Champagne F. A. D’Alessio A. C. Sharma S. Seckl J. R.. (2004). Epigenetic programming by maternal behavior. Nat. Neurosci. 7, 847–854. 10.1038/nn1276 15220929

[B228] Weaver I. C. G. Champagne F. A. Brown S. E. Dymov S. Sharma S. Meaney M. J.. (2005). Reversal of maternal programming of stress responses in adult offspring through methyl supplementation: altering epigenetic marking later in life. J. Neurosci. 25, 11045–11054. 10.1523/JNEUROSCI.3652-05.2005 16306417 PMC6725868

[B229] Wei L. Hao J. Lacher R. K. Abbott T. Chung L. Colangelo C. M.. (2015). Early-life stress perturbs key cellular programs in the developing mouse hippocampus. Dev. Neurosci. 37, 476–488. 10.1159/000430861 26068561 PMC4644446

[B230] Wei L. Simen A. Mane S. Kaffman A. (2012). Early life stress inhibits expression of a novel innate immune pathway in the developing hippocampus. Neuropsychopharmacology 37, 567–580. 10.1038/npp.2011.239 21993208 PMC3242319

[B231] Williams B. P. Price J. (1995). Evidence for multiple precursor cell types in the embryonic rat cerebral cortex. Neuron 14, 1181–1188. 10.1016/0896-6273(95)90265-1 7605631

[B232] Williams B. P. Park J. K. Alberta J. A. Muhlebach S. G. Hwang G. Y. Roberts T. M.. (1997). A PDGF-regulated immediate early gene response initiates neuronal differentiation in ventricular zone progenitor cells. Neuron 18, 553–562. 10.1016/s0896-6273(00)80297-4 9136765

[B233] Winkelmann-Duarte E. C. Padilha-Hoffmann C. B. Martins D. F. Schuh A. F. S. Fernandes M. C. Santin R.. (2011). Early-life environmental intervention may increase the number of neurons, astrocytes and cellular proliferation in the hippocampus of rats. Exp. Brain Res. 215, 163–172. 10.1007/s00221-011-2881-y 21969209

[B234] Winkler Z. Kuti D. Ferenczi S. Gulyás K. Polyák Á. Kovács K. J. (2017). Impaired microglia fractalkine signaling affects stress reaction and coping style in mice. Behav. Brain Res. 334, 119–128. 10.1016/j.bbr.2017.07.023 28736330

[B235] Wlodarczyk A. Holtman I. R. Krueger M. Yogev N. Bruttger J. Khorooshi R.. (2017). A novel microglial subset plays a key role in myelinogenesis in developing brain. EMBO J. 36, 3292–3308. 10.15252/embj.201696056 28963396 PMC5686552

[B236] Woolley C. S. Gould E. McEwen B. S. (1990). Exposure to excess glucocorticoids alters dendritic morphology of adult hippocampal pyramidal neurons. Brain Res. 531, 225–231. 10.1016/0006-8993(90)90778-a 1705153

[B237] Wozniak J. R. Riley E. P. Charness M. E. (2019). Clinical presentation, diagnosis and management of fetal alcohol spectrum disorder. Lancet Neurol. 18, 760–770. 10.1016/S1474-4422(19)30150-4 31160204 PMC6995665

[B238] Xiong J. Y. Li S. C. Sun Y. X. Zhang X. S. Dong Z. Z. Zhong P.. (2015). Long-term treadmill exercise improves spatial memory of male APPswe/PS1dE9 mice by regulation of BDNF expression and microglia activation. Biol. Sport 32, 295–300. 10.5604/20831862.1163692 26681831 PMC4672160

[B239] Yang J. Yang H. Liu Y. Li X. Q28n L. Lou H.. (2016). Astrocytes contribute to synapse elimination *via* type 2 inositol 1,4,5-trisphosphate receptor-dependent release of ATP. eLife 5:e15043. 10.7554/eLife.15043 27067238 PMC4829431

[B240] Ye F. Chen Y. Hoang T. Montgomery R. L. Zhao X. H. Bu H.. (2009). HDAC1 and HDAC2 regulate oligodendrocyte differentiation by disrupting the β-catenin-TCF interaction. Nat. Neurosci. 12, 829–838. 10.1038/nn.2333 19503085 PMC2701973

[B241] Zaychik Y. Fainstein N. Touloumi O. Goldberg Y. Hamdi L. Segal S.. (2021). High-intensity exercise training protects the brain against autoimmune neuroinflammation: regulation of microglial redox and pro-inflammatory functions. Front. Cell. Neurosci. 15:640724. 10.3389/fncel.2021.640724 33708074 PMC7940666

[B242] Zeng H. Zhang X. Wang W. Shen Z. Dai Z. Yu Z.. (2020). Maternal separation with early weaning impairs neuron-glia integrity: non-invasive evaluation and substructure demonstration. Sci. Rep. 10:19440. 10.1038/s41598-020-76640-y 33173142 PMC7656452

[B243] Zerlin M. Levison S. W. Goldman J. E. (1995). Early patterns of migration, morphogenesis and intermediate filament expression of subventricular zone cells in the postnatal rat forebrain. J. Neurosci. 15, 7238–7249. 10.1523/JNEUROSCI.15-11-07238.1995 7472478 PMC6578041

[B244] Zetter M. A. Hernández V. S. Roque A. Hernández-Pérez O. R. Gómora M. J. Ruiz-Velasco S.. (2021). Microglial synaptic pruning on axon initial segment spines of dentate granule cells: Sexually dimorphic effects of early-life stress and consequences for adult fear response. J. Neuroendocrinol. 33:e12969. 10.1111/jne.12969 33890333 PMC13186562

[B245] Zhan Y. Paolicelli R. C. Sforazzini F. Weinhard L. Bolasco G. Pagani F.. (2014). Deficient neuron-microglia signaling results in impaired functional brain connectivity and social behavior. Nat. Neurosci. 17, 400–406. 10.1038/nn.3641 24487234

[B246] Zhou Q. Wang S. Anderson D. J. (2000). Identification of a novel family of oligodendrocyte lineage-specific basic helix-loop-helix transcription factors. Neuron 25, 331–343. 10.1016/s0896-6273(00)80898-3 10719889

